# Genome-Wide Association Study Identifies Loci for Body Composition and Structural Soundness Traits in Pigs

**DOI:** 10.1371/journal.pone.0014726

**Published:** 2011-02-24

**Authors:** Bin Fan, Suneel K. Onteru, Zhi-Qiang Du, Dorian J. Garrick, Kenneth J. Stalder, Max F. Rothschild

**Affiliations:** 1 Department of Animal Science and Center for Integrated Animal Genomics, Iowa State University, Ames, Iowa, United States of America; 2 Key Laboratory of Agricultural Animal Genetics, Breeding and Reproduction, Ministry of Education & College of Animal Science and Technology, Huazhong Agricultural University, Wuhan, People's Republic of China; Institute of Preventive Medicine, Denmark

## Abstract

**Background:**

The recent completion of the swine genome sequencing project and development of a high density porcine SNP array has made genome-wide association (GWA) studies feasible in pigs.

**Methodology/Principal Findings:**

Using Illumina's PorcineSNP60 BeadChip, we performed a pilot GWA study in 820 commercial female pigs phenotyped for backfat, loin muscle area, body conformation in addition to feet and leg (FL) structural soundness traits. A total of 51,385 SNPs were jointly fitted using Bayesian techniques as random effects in a mixture model that assumed a known large proportion (99.5%) of SNPs had zero effect. SNP annotations were implemented through the Sus scrofa Build 9 available from pig Ensembl. We discovered a number of candidate chromosomal regions, and some of them corresponded to QTL regions previously reported. We not only have identified some well-known candidate genes for the traits of interest, such as MC4R (for backfat) and IGF2 (for loin muscle area), but also obtained novel promising genes, including CHCHD3 (for backfat), BMP2 (for loin muscle area, body size and several FL structure traits), and some HOXA family genes (for overall leg action). The candidate regions responsible for body conformation and FL structure soundness did not overlap greatly which implied that these traits were controlled by different genes. Functional clustering analyses classified the genes into categories related to bone and cartilage development, muscle growth and development or the insulin pathway suggesting the traits are regulated by common pathways or gene networks that exert roles at different spatial and temporal stages.

**Conclusions/Significance:**

This study is one of the earliest GWA reports on important quantitative traits in pigs, and the findings will contribute to the further biological function analysis of the identified candidate genes and potential utilization of them in marker assisted selection.

## Introduction

The domestic pig has a long history of extensive natural and artificial selection partly to meet human dietary needs [1]. In the past, conventional artificial selection relying on phenotype and pedigree information has been practiced for genetic improvement. However, human population growth is increasing rapidly and nutritional input from animal industries will be required to feed a hungry world. Thus, to further increase the rate of genetic improvement, understanding of the interplay between polygenic and environmental factors controlling complex agriculturally important production and disease-resistance traits is needed [2]. This information could be integrated in marker-assisted selection (MAS) schemes to increase selection accuracy, shorten generation interval, and accelerate genetic improvement.

Both candidate gene and QTL mapping strategies have been used in domestic animals for the discovery of genetic markers suitable for MAS [3]. To date, more than 5,500 QTL relevant to ∼550 overlapping phenotypic traits have been deposited in pig QTLdb (http://www.animalgenome.org/cgi-bin/QTLdb/SS/index). However, those approaches have limitations. Candidate gene selection according to their putative physiological roles could be limited, and may miss novel gene identification and / or pathways influencing the traits. The regions of identified QTL are generally large and fine mapping is required to find more closely linked markers or causative variants suitable for marker-assisted selection. The consistency of QTL mapping may be limited when it is based among resource families developed from diverse founders [3]. However, the recently completed genome sequencing projects in many species and newly developed high density SNP arrays have made it possible to conduct GWA and genomic selection studies for several species of food producing animals, which has opened a new era for animal breeding [4]–[7].

In contrast to studies in humans where association analyses have the primary purpose of identifying markers for disease, the applications of dense SNP arrays in livestock focus on genomic selection (also termed genomic prediction or genomic evaluation), in order to improve selection accuracy to accelerate genetic improvement for economically important performance traits in breeding animals [8]–[10]. Assuming abundant availability of SNPs scattered throughout the genome which can capture linkage disequilibrium (LD) relationships with QTL, Meuwissen and colleagues [8] proposed a novel genomic selection concept, *i.e*., to predict an animal's breeding values using information provided by numerous SNPs across the entire genome. In this seminal work, two Bayesian approaches (Bayes A and Bayes B) were developed to predict genomic estimated breeding values (GEBV). In the years following this paper, alternative statistical approaches for genomic selection have been developed, derived from Bayesian models and some other parametric methods including GBLUP and mixed regression models [11]–[14]. This genomic selection methodology can be feasibly adapted for a GWA study and in the context of mixture models genomic regions and SNPs having high model frequency (*i.e*., frequently included in the model for GEBV prediction) can be identified as those likely linked to QTL [8], [13], [15].

Most of the published GWA studies carried out in animals involved Mendelian or disease related traits [16]–[20], although some evaluated single marker mixed model associations for growth traits [21], [22]. Few GWA studies using Bayesian methods that concurrently fit many or all SNPs have yet been reported.

Loin muscle depth or loin area and fat depth are good predictors of carcass lean content and are two traits targeted in pig breeding programs. Body conformation as well as feet and leg (FL) structural soundness are relevant to growth, feed intake capability, reproductive efficiency and sow longevity and are receiving more attention in modern swine production where pigs are being raised in confined production systems. It has been reported that approximately 40% of breeding sows were culled because of FL problems [23], [24]. The objective of this study was to perform a GWA study with the porcine 60K SNP BeadChip and to identify candidate SNPs/genes and chromosomal regions associated with growth, body composition, body conformation and FL structural traits, that might be suitable for MAS and genomic selection. To our knowledge, this is the first report from a GWA study on economically important production traits in pigs. Furthermore, it will contribute to a better understanding of the genetic control of complex animal agriculture traits.

## Results

### Phenotype statistics

Detailed descriptions of exploratory analyses of all phenotypes are in Tables S1 and S2. Apart from four traits in which the preferred value is intermediate, such as top line, weak/upright legs, front and rear legs turned in/out, population average values of body conformation and FL structure traits were between 2.19 and 5.39. The means for uneven front/rear toes were approximately 2.2, indicating small inside toe conformation was not a problem in the studied population. The number of animals with front/rear legs turned out was rare, so only traits for front/rear legs turned in were analyzed. Distribution analysis found that most of the traits apparently follow or nearly follow normal distributions (data not shown).

### SNP chip data evaluation

The current Porcine 60K Beadchip has 64,232 SNPs [5], and based on the current pig genome annotation, *Sus scrofa* (SSC) Build 9, a total of 55,446 of these SNPs have been mapped to a genomic location. The average physical distance between any two neighboring SNPs on the same chromosome was approximately 41.6 Kb, ranging from 35.2 Kb (SSC14) to 81.4 Kb (SSCX) (Table S3). Based on the length of each chromosome in the USDA-MARC v2 (A) linkage map (http://www.thearkdb.org), the average genetic distance between SNPs on the chip is 0.046 cM, ranging from 0.02 cM (SSC1) to 0.08 cM (SSCX) (Table S3).

The distribution characteristics of SNPs were analyzed and among mapped loci, 15,338 are intragenic and are from 7,099 genes. The maximum number of SNPs from one gene is 33 and it is the *PARK2* gene located between 6.5 and 7.5 Mb on SSC1.

Only five genotyped animals had average genotype call rates less than 80%, and were removed from further analysis. Additionally, SNPs with no call (1,886) or call rates less than 90% (400) were discarded. A total of 51,385 SNPs passed these quality control steps and were retained in the dataset.

### GWA analysis

A population stratification analysis using IBS distance clustering showed that gilts from the two lines present in the data could be classified into one population, suggesting no significant genetic difference existed between these two lines. Since pedigree information indicated the lines had been separated, lines were fitted as a fixed effect in the model for association analyses.

Based on the heritability of each trait (Table S2), appropriate priors for the genetic and residual variances were obtained for input into the Bayesian statistical models. Results from the analyses including mean, posterior genetic and residual variances, and the resultant proportion of variation accounted for by SNPs are in Table S2. The genetic contribution to variation was relatively high for some traits for instance, 10^th^ rib backfat with around 250 SNPs likely accounting for 54% of total genetic variance (µ = 0.995), loin muscle area with the same number of SNPs accounting for 55%. While other traits, such as weak top line and high top line, had relatively lower heritability estimates.

The putative candidate chromosomal regions were obtained by finding those genomic locations comprising windows of 5 consecutive SNPs with the highest genetic variance. Significance level was subsequently determined by bootstrap analyses under the null hypothesis that these windows did not harbor QTL. The model frequency statistic was used to find the most promising SNPs within these regions. With the assumption that the experiment had 50% power and a 99% null hypothesis of no QTL in the SNP window, then the PFP (proportion of false positives) from Fernando *et al.* [25] was 0.66 for p<0.01 and 0.16 for p<0.001. Therefore, we expected at least half of the reported QTL to be real for all the traits studied in the present work.

Analyses for two backfat traits, the last rib backfat and 10^th^ rib backfat revealed that some of the genes underlying them were different, even though their genetic correlation has been estimated to be nearly 0.90 [26]. However, they did share several common regions, including those containing *MC4R* on SSC1, *ATP6V1H* and *OPRK1* genes on SSC4, *LDHD* gene on SSC6, *CHCHD3* gene on SSC18 and *ATP2B3* genes on SSCX. In total, 21 regions were associated with last rib backfat (Figure S2), and genes in these regions are listed in Table S4.

**Figure 1:**
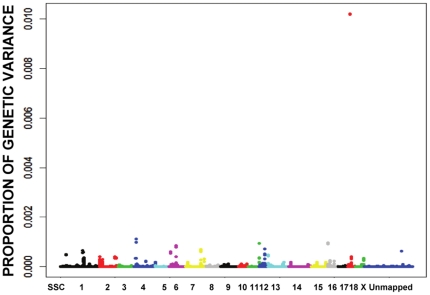
Proportion of genetic variance explained by each window of 5 consecutive SNP markers across the genome for 10th rib backfat, which was used to determine the candidate genome regions surrounding the significant SNPs. The X-axis is SNP marker position in genome order, and the Y-axis represents genetic variance of 5-SNP window (the exact candidate regions, the most promising SNPs and P values are shown in Table 1). Different colors represent SNPs on different chromosomes from SSC1 to X and unmapped markers.

As for 10^th^ rib backfat, 18 candidate regions contributing the largest genetic variance were identified and they were distributed along chromosomes 1, 2, 4, 6, 7, 11, 12, 16, 18 and X (Figure 1). Surprisingly, one novel region was identified by the SNP ALGA0118164 on SSC18, with a high model frequency of 0.63, while others were under 0.25 (Figure S3). A larger population size will be required to verify the significance of this SNP since it represents a novel QTL region. Of these 18 peaks, 9 corresponded to QTL regions associated with 10^th^ rib backfat in previous reports (Table S5). For example, the peak comprising four SNPs (INRA0004898, ALGA0006599, ALGA0006623 and ASGA0005017) on SSC1 was in a QTL region, where a known causative gene *MC4R* associated with fat and growth rate is located [27]. The peak pertaining SNP M1GA0005986 on SSC4 was close to a QTL region reported in the first pig QTL mapping study [28]. The genes within the regions were examined via pig Ensembl (http://www.ensembl.org/Sus_scrofa/Info/Index), and are listed in Table S5, together with those involved in fat development.

**Figure 2:**
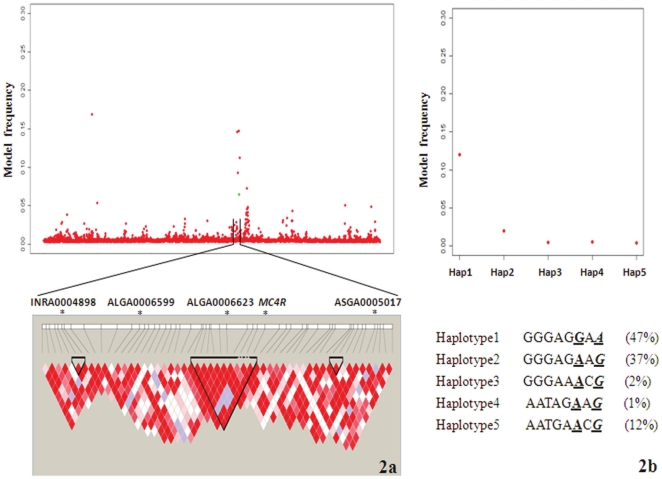
Haplotype analysis. **a)** The haplotype blocks of the region between 165.47–168.63 Mb on SSC1 where four SNPs (INRA0004898, ALGA0006599, ALGA0006623 and ASGA0005017) with model frequency greater than 0.10 for 10th rib backfat and the *MC4R* gene (green color dot) were located , and **b)** The plots of model frequency for each haplotype containing the SNPs ALGA0006623 (underlined) and *MC4R* Asp298Asn (Italicized and underlined).

The SNP *MC4R* Asp298Asn along with any other SNP involved in previous patents or patent applications were not included in the chip so that no infringement would exist. However, the SNP MC4R Asp298Asn was evaluated in this population in an earlier study [29]. Genotypes of *MC4R* Asp298Asn were added to the dataset, and fitted as a random effect in a Bayes C model, achieving a model frequency of 0.08 (Figure 2a) with the average effect of allele A being 2.74E-03, implying allele A (Asn298) was associated with increased fat thickness, in agreement with Kim *et al* [27]. When *MC4R* Asp298Asn was fitted as a fixed effect, the model frequencies for other SNPs (INRA0004898, ALGA0006599, ALGA0006623 and ASGA0005017) dropped, suggesting there may be interactions associated with backfat between *MC4R* and other SNPs in the region.

Haplotype analysis was carried out on a 2.5 Mb region containing the *MC4R* Asp298Asn SNP and four other significant SNPs. The other SNPs were in different haplotype blocks, whereas ALGA0006623 and Asp298Asn were from the same block (Figure 2a). The five major haplotypes with occurrence frequency great than 1% were assigned to each individual and these covariates merged into the SNP dataset, and each haplotype model frequency was obtained (Figure 2b). Compared to that of single SNPs, the haplotype model frequency did not increase much. It suggested that there may be other genes associated with backfat besides *MC4R* in this region. Similar results have been reported previously [30], and further studies are needed to identify additional candidates.

**Figure 3:**
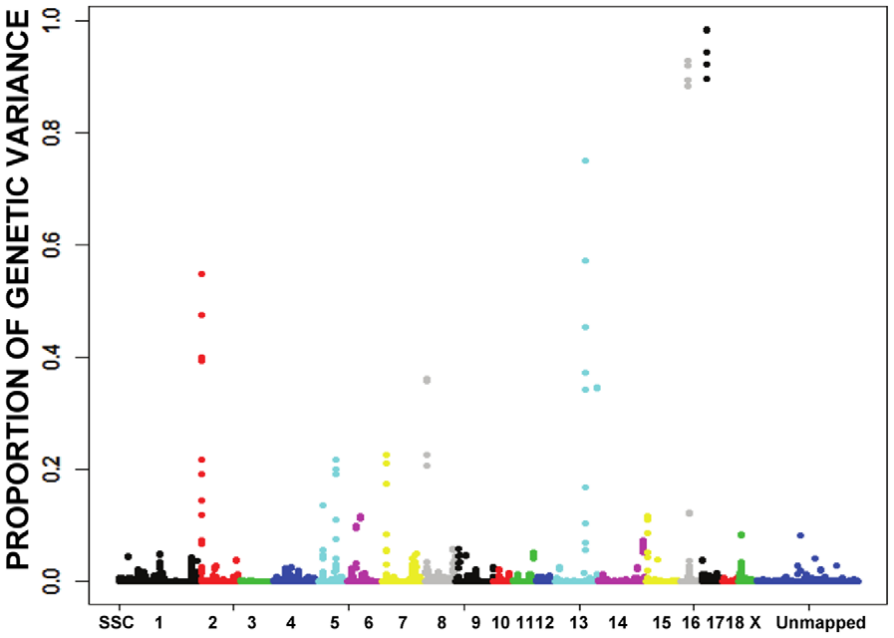
Proportion of genetic variance explained by each window of 5 consecutive SNP markers across the genome for 10th rib loin muscle area, which was used to determine the candidate genome regions surrounding the significant SNPs. The X-axis is SNP marker position in genome order, and the Y-axis represents genetic variance of 5-SNP window (the exact candidate regions, the most promising SNPs and P values are shown in Table S6). Different colors represent SNPs on different chromosomes from SSC1 to X and unmapped markers.

Loin muscle area is related to muscle development, and 14 peaks were associated with this trait and the significance level of 13 of those candidate regions reached 0.05 (Figures 3 and S4). Eight of 14 peaks corresponded to previously reported QTL regions for LMA. The most significant SNP, INRA0052808 was within the *BMP2* gene on SSC17, and the next two most significant SNPs, M1GA0002180 and M1GA0002244, were from the beginning of SSC2, where the *IGF2* gene is located. Furthermore, in the region including ALGA0090171on SSC16, there were two potentially interesting genes, *FST* and *NDUFS4* (Table S6).

The genetic variance contributed by SNP windows were plotted against genomic location for body size (body length, depth and width) and body shape traits (correctness of top line, rib shape and hip structure) and are shown in Figures S6a-e, with genes from the candidate regions being listed in Table S7. There were a few common candidate regions over these body conformation traits. The *BMP2* gene was associated with three body size traits, and interestingly, one allele was associated with long body length, shallow body and narrow body width (Table 1). The region at 270 Mb on SSC1 was also important for body size, where the *PAPPA* gene was considered as a candidate gene.

**Table 1:** The SNPs within *BMP_2_* genes associated with multiple traits, being estimated through a Bayesian procedure.

SNP	Trait (Unit)	Model frequency	Marker effect	Marker effect Delta	P value
MARC0070553	Body length	0.274	0.028	0.104	<0.001
	Body depth	0.296	0.043	0.145	<0.001
	Body width	0.080	−0.008	−0.104	<0.001
	Front leg pastern	0.295	0.061	0.206	<0.001
	Rear leg pastern	0.019	0.001	0.071	<0.001
	Rib shape	0.235	0.036	0.152	<0.001
	Buck knee	0.129	0.020	0.156	<0.001
	Loin muscle area (cm^2^)	0.035	−0.018	−0.529	<0.001
INRA0052808	Body length	0.299	0.033	0.110	<0.001
	Body depth	0.825	0.157	0.190	<0.001
	Body width	0.119	−0.014	−0.115	<0.001
	Front leg pastern	0.146	0.029	0.198	<0.001
	Rear leg pastern	0.074	0.008	0.111	<0.001
	Rib shape	0.154	0.023	0.149	<0.001
	Buck knee	0.034	0.004	0.124	<0.001
	Loin muscle area (cm^2^)	0.407	−0.323	−0.792	<0.001

Marker effect defines the posterior mean of the covariate effect averaged across the post-burnin chain. Marker effect Delta defines the posterior mean effect for only those chains that included the effect in the model, *i.e.*, Marker Effect/ModelFreq. P values indicate the significant confidence of candidate regions containing the analyzed SNPs, which were determined the genetic variance of 5-SNPs sliding window.

**Figure 4:**
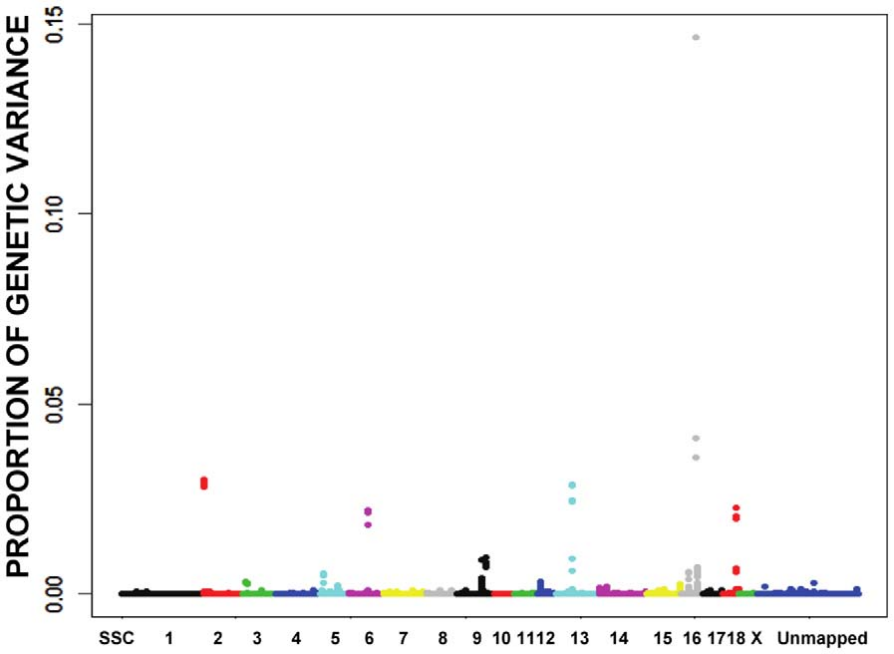
Proportion of genetic variance explained by each window of 5 consecutive SNP markers across the genome for overall leg action, which was used to determine the candidate genome regions surrounding the significant SNPs. The X-axis is SNP marker position in genome order, and the Y-axis represents genetic variance of 5-SNP window (the exact candidate regions, the most promising SNPs and P values are shown in Table S8). Different colors represent SNPs on different chromosomes from SSC1 to X and unmapped markers.

For overall leg action, there were 14 candidate regions (Figures 4 and S5), which are from chromosomes 2, 3, 5, 6, 9, 13, 15, 16 and 18 (Table S8). The most significant region included SNPs H3GA0046827 and H3GA0046828 on SSC16, where no coding gene has been identified. In the candidate region on SSC2 surrounding MARC0022036, there was no gene with biological significance for skeletal development and locomotion. On SSC18, the region including HOX genes (*HOXA1, HOXA2* and *HOXA3*) was identified, and on SSC9, the region containing the *TWIST1* and *SP4* genes was detected.

However, for FL structural traits, which have relatively lower heritabilities, multiple genes seem to be involved. The putative candidate genes associated with traits were quite different, even for similar traits such as front leg pastern and rear leg pastern positions, and different traits at the same body position such as front leg pastern and front leg turned in (Figure S7 and Table S9). A clustering analysis for genes based on their functional annotation demonstrated that they could be classified into different categories, which related to the original traits. Some could be involved in bone and cartilage development such as *SOX9, LRCH1, BMP2, COL4A3* and *COL4A4*, some might be related to muscle development such as *MYOD1, MUSK, MYH1, MYH2, MYL9*, *MAP2K6* and *MAP3K4*, while others could be relevant to the insulin pathway such as *PDX1, PTPN1, CTGFL* and *WISP2*. These groups are all known to be associated with growth and development.

## Discussion

The GWA and genomic selection studies conducted to date are beginning a promising era in animal breeding. To our knowledge, this might be the earliest GWA report on economically important growth and production traits in pigs. The primary results will contribute to the dissection of molecular mechanisms regulating important quantitative traits, identification of novel candidate genes and networks, and functional analysis of promising genes.

It is likely that GWA studies will be increasingly important to animal improvement. For disease related traits, a case-control strategy that is well-known in human studies has been generally utilized [16], [19]. For quantitative traits, several different methods have been explored [10], [18], [20]. In the present study a newly developed approach for GWA analyses between SNPs and quantitative traits was evaluated. In comparison to Bayes B, this approach was less reliant on priors for the genetic and residual variance, and computational time was reduced.

Population stratification is a confounding component that affects association analyses. The animals in this study were from two different genetic lines, which originated from the same breed resources. A clustering analysis based upon IBS distance classified them into one population. Nevertheless, the lines were fitted as a fixed effect in the model for association analyses based on pedigree information.

The Bayesian method used for association analyses in this study has been demonstrated to have higher accuracy even with different relationship scenarios between training and validation populations [31], [32]. It indicated that association results from the two lines in this study were robust and could be suitable for MAS and genomic selection in Large White and related pig populations.

A total of 18 candidate chromosomal regions were identified for 10^th^ rib backfat, and half of them corresponded to previously reported QTL regions. The most promising SNP ALGA0118164 was 13 kb upstream of the *CHCHD3* (coiled-coil-helix-coiled-coil-helix domain containing 3) gene, which is primarily expressed in mitochondria. The *Leptin* gene is around 4 Mb downstream of this gene, but the nearest SNP M1GA0023128 to *Leptin* was not in strong LD with ALGA0118164 and it did not achieve high model frequency (0.004). There are no reports about the association between *CHCHD3* and phenotypes related to obesity/diabetes in humans and rodents. An investigation involving additional SNPs within *CHCHD3* and functional analysis using adipocyte lines is warranted to verify the association of this gene with fat development.

One important region was identified between 165.47–168.63 Mb on SSC1, with around 20 coding genes annotated in *Sus scrofa* Build 9, including *MC4R,* one of the important functional genes associated with backfat. In pigs, the SNP Asp298Asn was found to be significantly associated with backfat and growth rate [27], and the association with fat has been implicated in pigs with diverse backgrounds. Four SNPs (INRA0004898, ALGA0006599, ALGA0006623 and ASGA0005017) distributed in different haplotype blocks were studied. The model frequency values decreased for the other SNPs when any one of them was considered as a fixed instead of random effect (data was not shown). The genes *BCL2* and *CCBE1* are two of interest. In addition, the candidate region harboring the SNP M1GA0005986 on SSC4 corresponded to a fat QTL location reported in the first porcine QTL mapping study [28]. Berg *et al.* [33] characterized this FAT1 QTL and refined its map position to a 3.3 cM interval between the *RXRG* and *SDHC* genes. These two genes are located at 89 Mb on SSC4 (Build 9), about 8 Mb from the region we found. The actual FAT1 QTL effects caused by the identified genes or others such as *ATP6V1H* and *OPRK1* in this region should be explored to identify the causative mutation.

There were 14 candidate regions associated with LMA, and eight of them corresponded to QTL regions previously reported. The most interesting SNP INRA0052808 was from the *BMP2* gene on SSC17. It is well known that *BMP2* is involved in regulating early myogenesis and could inhibit proliferation or induce presumptive muscle cells to undergo apoptosis, thus inhibiting muscle development. These results, combined with those discussed later in this manuscript involving the association between *BMP2* and body dimensions help explain the role this gene has in controlling body dimension and muscle development. A region on SSC2 including the significant SNPs M1GA0002180 and M1GA0002244 is close to the locus where the *IGF2* gene is located. *IGF2* has been considered to be one of the major causative genes associated with muscle development [34], and the G3072A mutation in intron 3 has been proposed to cancel *in vitro* interaction with a nuclear factor and was associated with a threefold increase in *IGF2* messenger RNA expression in postnatal muscle [35].

A total of 14 candidate regions were found to be associated with overall leg action. On SSC18, one candidate region including a cluster of HOX family genes including *HOXA1, HOXA2* and *HOXA3* were identified. *HOX* genes encode a class of transcription factors that contain an antennapedia (HOM gene) related homeobox and are conserved throughout metazoan evolution in vertebrates, and they exert essential roles on the morphogenesis of skeletal structure along the antero-posterior axis [36]. On SSC6, a region including the *FHL3* gene was associated with leg action. *FHL3* negatively regulates myotube formation of C_2_C_12_ cells, and it could form a complex with MyoD inhibiting its transcription activity and control the expression of muscle specific genes such as muscle creatine kinase and myogenin [37]. Additionally, one region comprising three SNPs H3GA0027878, ALGA0054178 and ALGA0054186 on SSC9 was identified, and it contains the *TWIST1* and *SP4* genes, which are of interest for their association with leg action.

A number of candidate regions associated with body conformation traits were identified, but common genomic regions responsible for these traits appears to be rare. These results suggested that the genetic mechanisms underlying body growth and development are complicated, and the genes contributing to the body plan are in various networks and they might be regulated at different developmental stages, a fact known to biologists. The *BMP2* gene was associated with all three body size traits. The allele T of SNP INRA0052808 located in *BMP2* was associated with long body length, shallow body depth and narrow body width, and additionally it was associated with upright front/rear leg pastern postures and smaller LMA (Table 1). The genetic correlation between body length and body depth was positive while it was negative between these two traits and body width, and body width had a high favorable genetic correlation with LMA [26]. In practical breeding programs, animals could be selected as breeding stock based on the allele in BMP preferred by the breeder based on structural requirement.

There are a number of factors contributing to the FL structural soundness of livestock. The development of the body skeleton and muscle mass is an important factor determining body conformation, physical fitness and leg movement. In humans, the abnormal development of the skeleton can lead to a series of disorders, such as dwarfism, osteochondrosis, osteoporosis, osteopetrosis and osteoarthritis, which affect normal action capability and even result in lameness. Based on the functional clustering analyses we conducted, approximately 30% of candidate genes associated with FL could be classified into the categories of bone and cartilage development, skeletal muscle development and the insulin pathway. Some of the genes involved in bone and cartilage development were *SOX9, LRCH1, FBN2, COL4A3,* and *COL4A4*.

The genes and transcription factors *MYOD1, MuSK, MYH1, MYH2, MYL9*, *MAP2K6* and *MAP3K4* were found within candidate regions. Skeletal muscles are attached by tendons across joints, and muscle contraction drives the bones and joints. The muscle and skeleton function together and thus provide for animal movement as seen from a previous selection experiment [38], [39]. *MYOD1* is involved in muscle differentiation and can induce fibroblasts to differentiate into myoblasts [40]. Mutations in the *MuSK* gene may cause congenital myasthenic syndromes (CMS), a type of hereditary disease characterized by muscles that fatigue easily resulting in muscle weakness caused by neuromuscular transmission dysfunction in humans [41]. *MAP2K6* and *MAP3K4* are two of the MAPK kinases that are involved in the p38 mitogen-activated protein kinase (MAPK) pathway. The p38 MAPKs regulate the transcriptional activities of MRFs and function in the remodeling of chromatin at specific muscle-regulatory regions, and it is one of the major intracellular signaling pathways affecting myogenesis [42].

Both insulin and IGF1 are known to induce a wide variety of growth and metabolic responses and play essential roles in anabolic regulation of bone metabolism [43]. Several genes involved in the insulin and IGF1 pathway were identified to be associated with the FL traits in this study. *PDX1* could regulate the expression of pancreatic endocrine cell genes including insulin through the interaction at A-T rich regions contained within the promoter of these genes, and has a role in maintenance of the β-cell phenotype [44]. Mutations in the *PTPN1* promoter might contribute to the development of T2D and related metabolic traits [45]. *CTGFL* could promote the adhesion of osteoblast cells and inhibit the binding of fibrinogen to integrin receptors, and may play important roles in modulating bone turnover [46]. Associations of these genes with FL soundness traits need to be further verified.

In this study, a number of candidate chromosomal regions relevant to the analyzed traits were identified, and some were consistent with previously reported QTL regions. These new SNP chip results demonstrated that combining dense SNP arrays and using the Bayes C approach is ideal for GWA studies on important quantitative traits in domestic animals. Meanwhile, some issues are worth discussing when the findings were compared with those of conventional candidate gene studies [29]. Several promising candidate genes such as *PPARG,* Leptin receptor (*LEPR*) and *IGFBPs* (for backfat deposition), Myogenic regulatory factors (*MRFs*) and Myostatin (*MSTN*) (for muscle growth), and *CALCR*, *OXTR* and *PTHR* (for leg related traits), were not found to be associated with the analyzed traits in these animals. In addition, most of the traits - associated SNPs (TASs) were from novel genes without obvious biological significance relevant to the analyzed traits, and most of TASs were located in the intergenic regions or introns of coding genes. Similarly, in GWA studies of the humans, the TASs have not always been from the putative candidate genes relevant to disorders or diseases [47]. It has been reported that 88% of TASs were intronic (45%) or intergenic (43%), 9% were nonsynonymous, 2% were in a 5′ or 3′ untranslated regulatory regions and 2% was synonymous [48]. In dairy cattle, the suggested candidate genes underlying Johne's disease such as *SLC11A1*, *IL23R* and *NOD2* were not found to be associated with this disease [21]. In this study, we speculate that such discrepancies may have resulted from one of the following explanations: *i*) The TASs may be from genes that have not yet been annotated, or linked to the unmapped SNPs, therefore, further annotations on the current *Sus scrofa* Build 9 are necessary; *ii*) The sample size and the genetic backgrounds of the studied populations may have effects on association analyses, and additional pure breeds with larger sample size will be of help for GWA implications; *iii*) Although Bayesian methods are well suited for genomic selection and the statistics such as window variance and model frequency can be used to evaluate associations, the prediction accuracy of the methods were between 40–60%. Therefore, the indicator for association may not be the best one, and additional statistical approaches for GWA study are needed; *iv*) The large (∼40 Kb) average interval and uneven SNPs distributions of the current porcine 60 K BeadChip may be major limitations for haplotype blocks analyses and fine mapping [49], and a higher density SNP panel is worthy of being developed. Even with these limitations, the SNP chip and results presented here offer new opportunities to understanding the underlying genetic factors affecting these traits of interest.

## Materials and Methods

### Ethics statement

Animal care guidelines were followed according to the Institutional Animal Care and Use Committee (IACUC) at Iowa State University (Approval ID: log#7-05-5927-S).

### Animals and traits

A total of 820 gilts were included in the study, which were commercial breeding stock sourced from Newsham Choice Genetics (West Des Moines, IA, USA) between October 2005 and July 2006. The gilts belonged to two genetic lines and included 412 Large White line pigs and 408 pigs from a Large White×Landrace cross.

Body composition and structural soundness evaluations were carried out on 14 dates, with females having an average body weight of 124±11 kg and age of 190±7 days at appraisal time. Body composition traits comprised ultrasonically measured loin muscle area at the 10th rib, 10th rib backfat and last rib backfat. Ultrasonic images were taken with a Pie Medical 200 (Classic Medical Supply, Inc., Tequesta, FL) by a single technician certified by the National Swine Improvement Federation. A total of 17 body structural soundness traits were recorded including body conformation (body length, depth and width, top line, rib shape and hip structure), front feet and leg structure (legs turned, buck knees, pastern posture, foot size and uneven toes), rear feet and leg structure (legs turned, weak/upright legs, pastern posture, foot size and uneven toes) and overall leg action, which is a general appraisal reflecting both structural soundness and freedom of other defects affecting the gait and locomotion. All of the structural soundness traits were independently evaluated by two experienced scorers using nine-point scales, where one and nine indicated the extreme phenotypes of the traits. The intermediate score is the most favorable for four of the scoring traits including top line, turned front legs, turned rear legs and weak/upright rear legs. Scoring criterions and the descriptions of scores are shown in Table S1 and Figure S1, respectively.

### SNP array genotyping

A very small amount of ear tissue was collected from gilts using the TypiFix^TM^ ear tag from Agrobiogen (Hilgertshausen, Germany). The DNA was isolated from dry ear tissue using the DNeasy 96 Blood & Tissue Kit (Qiagen, Valencia, CA, USA). DNA quantification was performed using a NanoDrop 1000 (Thermo Fisher Scientific Inc., Wilmington, DE, USA). DNA samples were submitted for genotyping with total DNA between 700–1000 ng, 260/280 ratio>1.50 and DNA concentration>20 ng/ul. The genotyping was done by GeneSeek Inc. (Lincoln, NE, USA) using the Porcine 60K BeadChip (Illumina, San Deigo, CA, USA) and approved standard techniques outlined by the manufacturer. Quality control (QC) was performed after we received the original SNP genotyping data. The SNPs were filtered with call rate≥80%, Gentrain Score≥50%, minor allele frequency (MAF)≥0.05 and P value of 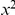 test for Hardy-Weinberg equilibrium≥1×E−6.

### Phenotypic statistics

The statistical analyses for descriptive analyses including population mean and normal distribution testing were performed using the UNIVARIATE procedure of the SAS software package (SAS Institute, release 9.2, Cary, NC, USA). Top line, turned front leg, turned rear legs and weak/upright rear legs were each divided into two traits prior to analyses because of intermediate optimum of the original trait scoring (Table S2). In addition, these separated traits (such as high top line and weak top line, front legs turned in and front legs turned out) had different heritability and moderate negative genetic corrections [33], implying that they are influenced by different genetic factors.

### Population stratification analysis

The animals in the study were from two genetic lines but originated from Large White or Large White×Landrace interbreed crossing. Population stratification was examined using an identical-by-state (IBS) distance clustering method in PLINK program [50].

### Bayesian methods for GWA analyzes

A new Bayesian method for genomic selection (Bayes C) has been proposed [15], developed from the Bayes B and GBLUP approaches [8]. The Bayes B approach is sensitive to the assumed prior of the genetic variance, and accuracy of genomic prediction could be affected if that value was given incorrectly. However, Bayes C is more tolerant to prior genetic variance, and is described here as follows: 
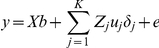
where *y* is the vector of phenotypes of the analyzed traits, *X* is the incidence matrix for fixed effects, 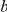 is the vector of fixed effects, *K* is the number of SNPs in the dataset, *Z_j_* is the column vector representing the SNP covariate at locus *j*, *u_j_* is the random substitution effect for locus *j*, which conditional on σ^2^ *_u_*, is assumed normally distributed N(0, σ^2^ *_u_*) when *δ_j_* = 1, while *u_j_* = 0, when *δ_j_* = 0, *δ_j_* is a random 0/1 variable indicating the absence (with probability π) or presence (with probability 1−π) of locus *j* in the model, and *e* is the vector of random residual effects assumed normally distributed N(0, σ^2^ *_e_*). Thus, both Bayes B and Bayes C are mixture models that assume a mixture of two distributions for the SNP effects, with assumed mixture fraction π. In these analyses, π was assumed to be 0.995. The variance σ^2^ *_u_* (or σ^2^ *_e_*) was a priori assumed to be scaled inverse chi-square with *v_u_* = 4 (or *v_e = _*10) degree of freedom and scale parameters *S* ^2^ *_u_* (or *S* ^2^ *_e_*) [51]. Additionally, in Bayes C σ^2^ *_u_* is common to all loci in the model in contrast to the locus specific variance component in Bayes B.

SNP effects *u_j_* were estimated using the Monte-Carlo means of the posterior distribution of these effects computed by a Gibbs sampling strategy. For a locus *j*, samples for *δ_j_* were obtained from its conditional distribution given b, the effects at all other loci included in the model and the two variance components σ^2^ *_u_* and σ^2^ *_e_*. This conditional distribution can be written as 
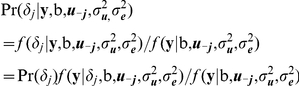
where ***u-_j_*** is the vector of effects in the model other than at locus *j*. The *f*(**y**| b, ***u-_j_*** , σ^2^ *_u_,* σ^2^ *_e_*) was obtained as the sum of Pr(*δ_j_*) *f*(**y**|*δ_j_*, b, ***u-_j_***, σ^2^ *_u_,* σ^2^ *_e_*) computed for *δ_j_*  = 0 and for *δ_j_*  = 1 [51].

In each iteration of a Monte Carlo Markov Chain, every SNP was subjected to a likelihood ratio test that determined the probability that SNP would be included in the model given the fixed effects and currently fitted markers, and that SNP was then included in the model in that iteration with the calculated probability. Evidence for an informative SNP was obtained by accumulating the frequency across iterations of the chain that a particular SNP was fitted in the model, we refer to this statistic as model frequency. If consecutive SNPs are in high linkage disequilibrium (LD) with the same QTL, then the effect of the QTL and the SNP model frequency will be distributed across all the SNPs in high LD with the QTL. For this reason model frequency of individual SNP is not a good indicator of the presence of QTL in genomic regions with high LD. Accordingly, two alternative approaches were used to infer presence of QTL. First, by accumulating the window model frequency whereby a genomic window of typically 5 immediately consecutive SNPs was counted as being included in the model when at least one of any of the SNPs in that window were in the model that iteration. Second, a QTL was inferred by computing a statistic representing the relative contribution of variation in window breeding value compared to variation in genomic breeding value. Window breeding value was computed by multiplying the number of Illumina B alleles that represent the SNP covariates for each consecutive SNP in a window by their respective posterior means for substitution effects. After computing these 5 SNP window breeding values for all animals in the population the variance of these breeding values was calculated. Windows that contributed the highest genetic variance were considered to be the strongest signals of association. Genomic breeding values are calculated using the same approach as window breeding values but utilized every SNP in the genome. These window approaches to identify important genomic regions accounted for linkage disequilibrium and better characterize QTL than individual SNP effects.

Several factors relevant to the traits, such as genetic line, measurement date and scorer, were considered as fixed class effects or covariates (e.g. body weight). The above procedures were implemented using a web-based program, GenSel (http://bigs.ansci.iastate.edu/bigsgui/login.html), developed by Fernando and Garrick [15].

### Bootstrap analysis

The calculation of the genetic variance accounted for by a 5 SNP window accounts for linkage disequilibrium between the 5 neighboring SNPs and discriminates important chromosomal effects from spurious effects of single SNPs. The chromosomal regions or the clusters of SNPs in sliding windows with the greatest contributions are also most likely to be associated with the analyzed phenotypes, and are considered to represent the QTL at that location. Using a similar approach with high density SNP chip data, Sun *et al* [52] recently identified QTL with few false positives for a complex pedigree containing QTLMAS 2010 data. That study also reported that the present genomic approach is at least as successful as other competing methods to identify QTL.

The significance level of the putative candidate genomic regions was estimated using bootstrap analysis. Bootstrap samples were produced using the posterior means of the 53,815 SNPs to construct the distribution of the test statistic (genetic variance of a SNP window) for each putative QTL. A bootstrap sample of the vector y for replicate *j* (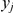) was created using the posterior means of the fixed 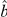 and SNP 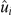 effects, except that all those SNP contained in the window that formed the QTL were excluded, and a vector of simulated residuals were added, formed by sampling a vector of independent standard normal deviations, 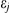 one deviation for each animal, scaled by the posterior mean of the residual standard deviation 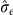, according to:
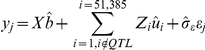



These bootstrap samples are constructed according to the null hypothesis of no QTL in the identified SNP window. Each bootstrap sample was reanalyzed using the Bayes C model used for the real data, and the genetic variance of the SNP window corresponding to the QTL were accumulated across all the bootstrap samples, for comparison to the test statistic represented by the genetic variance of the SNP window identified in the analysis of the real data. If just 1 bootstrap statistic from the 1,000 simulated exceeded the test statistic from the real data, the comparison-wise p-value was determined to be 0.001<p<0.002. In addition, multiple testing was taken in to account using the probability of false positives (PFP) as in Fernando *et al* [25]. That approach controls the probability of false positive conclusions across all the tests undertaken, rather than the probability of making one mistake over all tests as would be the interpretation of an experiment-wise error correction.

### Haplotype block and association analysis

Haplotype analysis was performed for the chromosomal regions where multiple candidate SNPs were located. The haplotype blocks were identified using Haploview (Ver 4.1) [53]. Haplotypes were obtained for each animal using the PHASE computer program (Ver. 2.1) [54], and for each individual, it carried 0, 1 and/or 2 copies of a certain haplotype. The association between the haplotypes and the traits were estimated using GenSel [15] as described above.

### Gene ontology

For regions harboring the SNP with the highest model frequency, gene search was performed via *Sus Scrofa* Build 9 of pig Ensembl (http://www.ensembl.org/Sus_scrofa/Info/Index). The QTL location for relevant traits and the physical positions of microsatellite markers retrieved from ArkDB (http://www.thearkdb.org) and PigQTL (http://www.animalgenome.org/cgi-bin/QTLdb/SS/index) were integrated, to obtain connections between QTL and candidate regions found from GWA studies.

Gene ontology analysis was performed to extract the functional annotation clustering using an online site called DAVID (http://david.abcc.ncifcrf.gov).

In addition, for those SNPs with high model frequency that were unassigned to any genomic location, their joint LD with SNPs of known location was calculated using *r^2^* so that the possible physical positions could be deduced from the SNPs with known location that had the highest estimated *r^2^* with the unassigned SNP.

## Supporting Information

Figure S1 The scoring criteria for body conformation, and feet and leg structure soundness traits.
(0.10 MB TIF)

Figure S2 Proportion of genetic variance explained by each window of 5 consecutive SNP markers across the genome for last rib backfat, which was used to determine the candidate genome regions surrounding the significant SNPs. The X-axis is SNP marker position in genome order, and the Y-axis represents accumulative genetic variance of 5-SNP window (the exact candidate regions, the most promising SNPs and P values are shown in Table S4). Different colors represent SNPs on different chromosomes from SSC1 to X and unmapped markers.
(0.07 MB TIF)

Figure S3 The SNP model frequency plots for assessing associations between markers and 10th rib backfat. The X-axis is SNP marker position in genome order, and the Y-axis represents model frequency (0.10 was considered as threshold for 10th rib back fat here). Different colors represent SNPs on different chromosomes from SSC1 to X and unmapped markers.
(0.08 MB TIF)

Figure S4 The plots of model frequency of SNPs for assessing the associations between the markers and 10th rib loin muscle area. The X-axis is SNP marker position in genome order, and the Y-axis represents model frequency (0.10 was considered as threshold for 10th rib loin muscle area here). Different colors represent SNPs on different chromosomes from SSC1 to X and unmapped markers.
(0.10 MB TIF)

Figure S5 The plots of model frequency of SNPs for assessing the associations between the markers and overall leg action. The X-axis is SNP marker position in genome order, and the Y-axis represents model frequency (0.05 was considered as threshold for overall leg action). Different colors represent SNPs on different chromosomes from SSC1 to X and unmapped markers.
(0.10 MB TIF)

Figure S6 Proportion of genetic variance explained by each window of 5 SNP consecutive markers across the genome for body conformation traits, which was used to determine the candidate genome regions surrounding the significant SNPs. The body conformation traits are a) body length; b) body depth; c) body width; d) rib shape and e) Hip structure. The X-axis is SNP marker position in genome order, and the Y-axis represents accumulative genetic variance of 5-SNP window (the exact candidate regions, the most promising SNPs and P values are shown in Table S7). Different colors represent SNPs on different chromosomes from SSC1 to X and unmapped markers.
(1.65 MB TIF)

Figure S7 Proportion of genetic variance explained by each window of 5 consecutive SNP markers across the genome for feet and leg structure soundness traits, which was used to determine the candidate genome regions surrounding the significant SNPs. The feet and leg structure soundness traits are a) front leg pastern; b) rear leg pastern; c) front buck knee; d) front feet size; e) rear feet size; f) front uneven toes and g) rear uneven toes. The X-axis is SNP marker position in genome order, and the Y-axis represents accumulative genetic variance of 5-SNP window (the exact candidate regions, the most promising SNPs and P values are shown in Table S9). Different colors represent SNPs on different chromosomes from SSC1 to X and unmapped markers.
(2.15 MB TIF)

Table S1 The description of the 17 analyzed traits of body conformation, feet and leg structure and overall leg action.
(0.04 MB DOC)

Table S2 The overall statistics about population means, estimated heritability and the genetic parameters of Bayes C analyses for the traits.
(0.05 MB DOC)

Table S3 The overall statistics on average interval between SNPs and SNP distribution of porcine 60K SNP array.
(0.05 MB DOC)

Table S4 The detail information about candidate regions and the most significant SNPs associated with last rib backfat.
(0.05 MB DOC)

Table S5 The detail information about the putative candidate regions and the most significant SNPs associated with 10th rib backfat.
(0.05 MB DOC)

Table S6 The detail information about the putative candidate regions and the most significant SNPs associated with 10th rib loin muscle area.
(0.05 MB DOC)

Table S7 The detail information about candidate regions and the most significant SNPs associated with body conformation traits.
(0.09 MB DOC)

Table S8 The detail information about the putative candidate regions and the most significant SNPs associated with overall leg action.
(0.05 MB DOC)

Table S9 The detail information about candidate regions and the most significant SNPs associated with feet and leg structural soundness traits.
(0.19 MB DOC)
