# Supplementary figures and images for: Genome-Wide Association Study Identifies Loci for Body Composition and Structural Soundness Traits in Pigs
Source: PLoS One. 2011 Feb 24;6(2):e14726. doi: 10.1371/journal.pone.0014726 (PMC3044704; doi:10.1371/journal.pone.0014726)

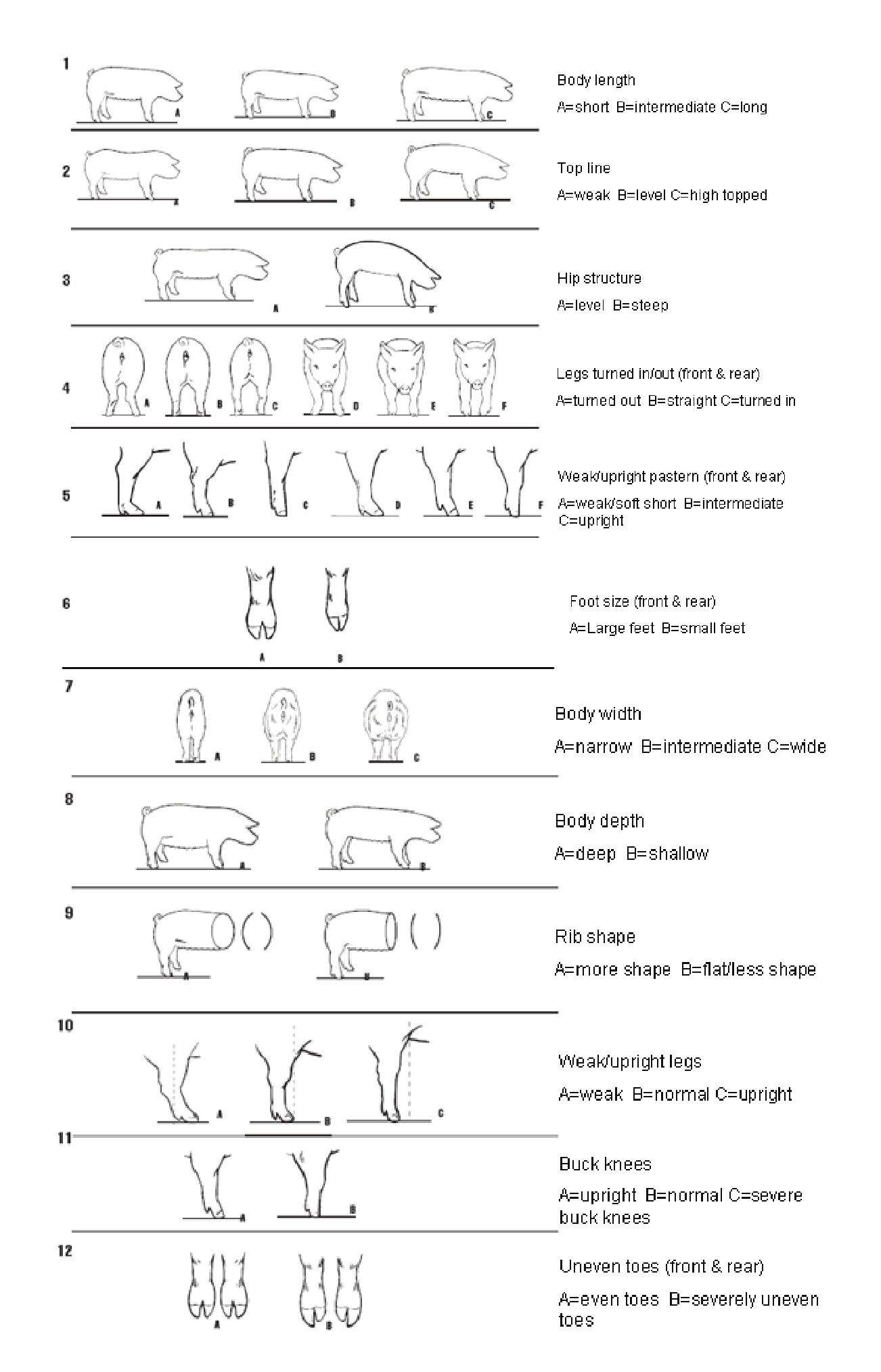

Supplement: Figure S1 — The scoring criteria for body conformation, and feet and leg structure soundness traits. (0.10 MB TIF) [file pone.0014726.s001.tif]

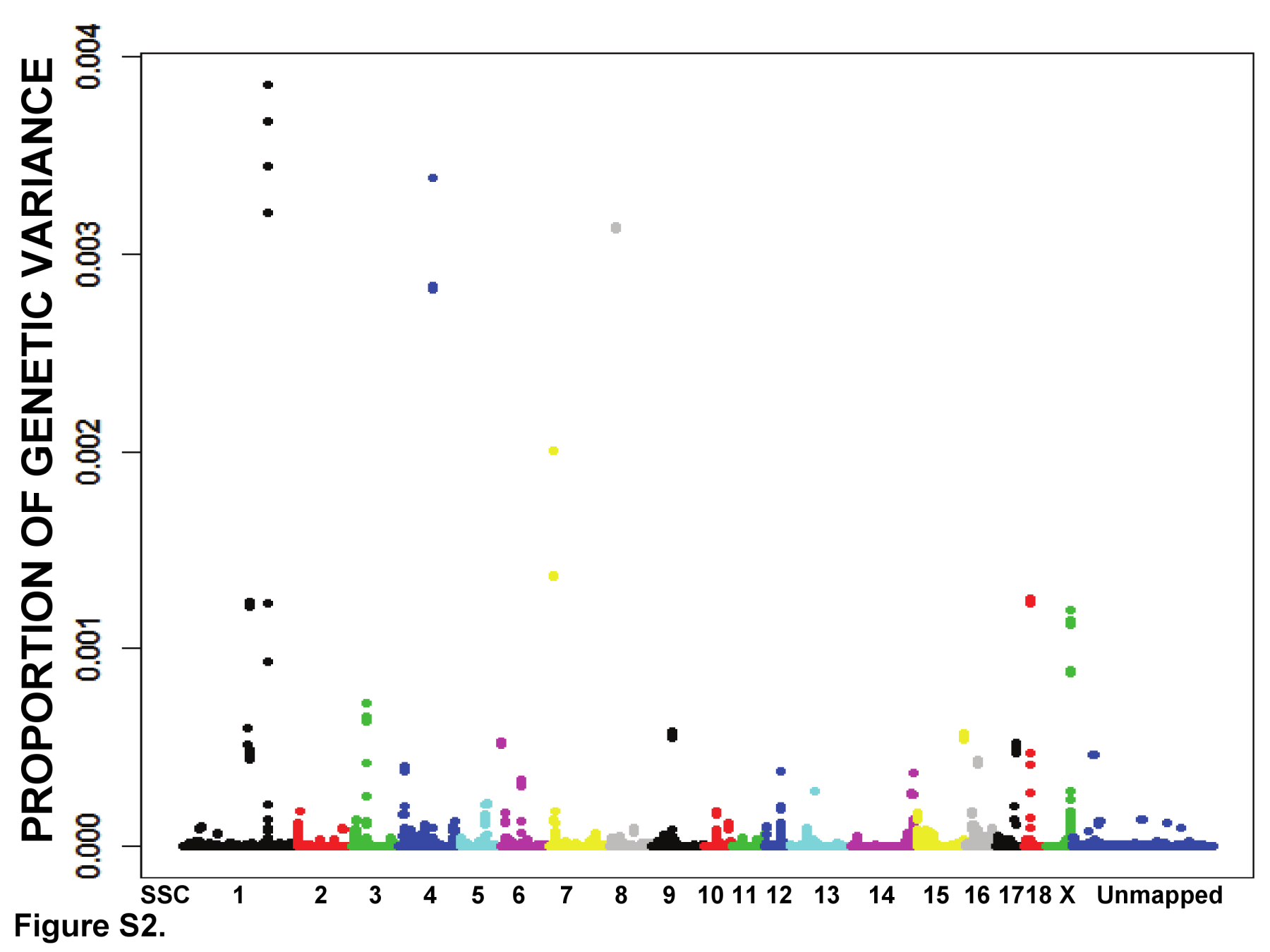

Supplement: Figure S2 — Proportion of genetic variance explained by each window of 5 consecutive SNP markers across the genome for last rib backfat, which was used to determine the candidate genome regions surrounding the significant SNPs. The X-axis is SNP marker position in genome order, and the Y-axis represents accumulative genetic variance of 5-SNP window (the exact candidate regions, the most promising SNPs and P values are shown in Table S4). Different colors represent SNPs on different chromosomes from SSC1 to X and unmapped markers. (0.07 MB TIF) [file pone.0014726.s002.tif]

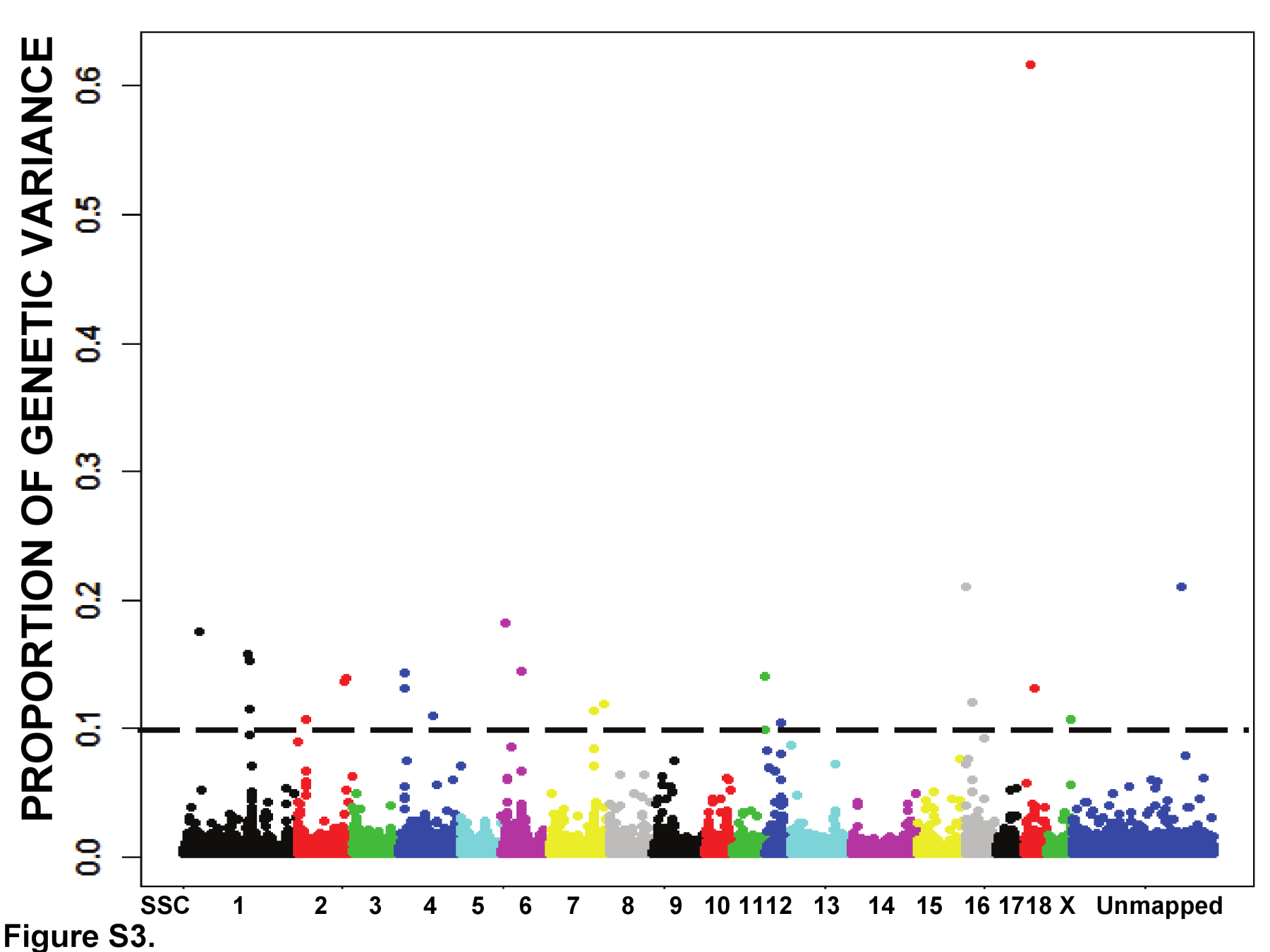

Supplement: Figure S3 — The SNP model frequency plots for assessing associations between markers and 10th rib backfat. The X-axis is SNP marker position in genome order, and the Y-axis represents model frequency (0.10 was considered as threshold for 10th rib back fat here). Different colors represent SNPs on different chromosomes from SSC1 to X and unmapped markers. (0.08 MB TIF) [file pone.0014726.s003.tif]

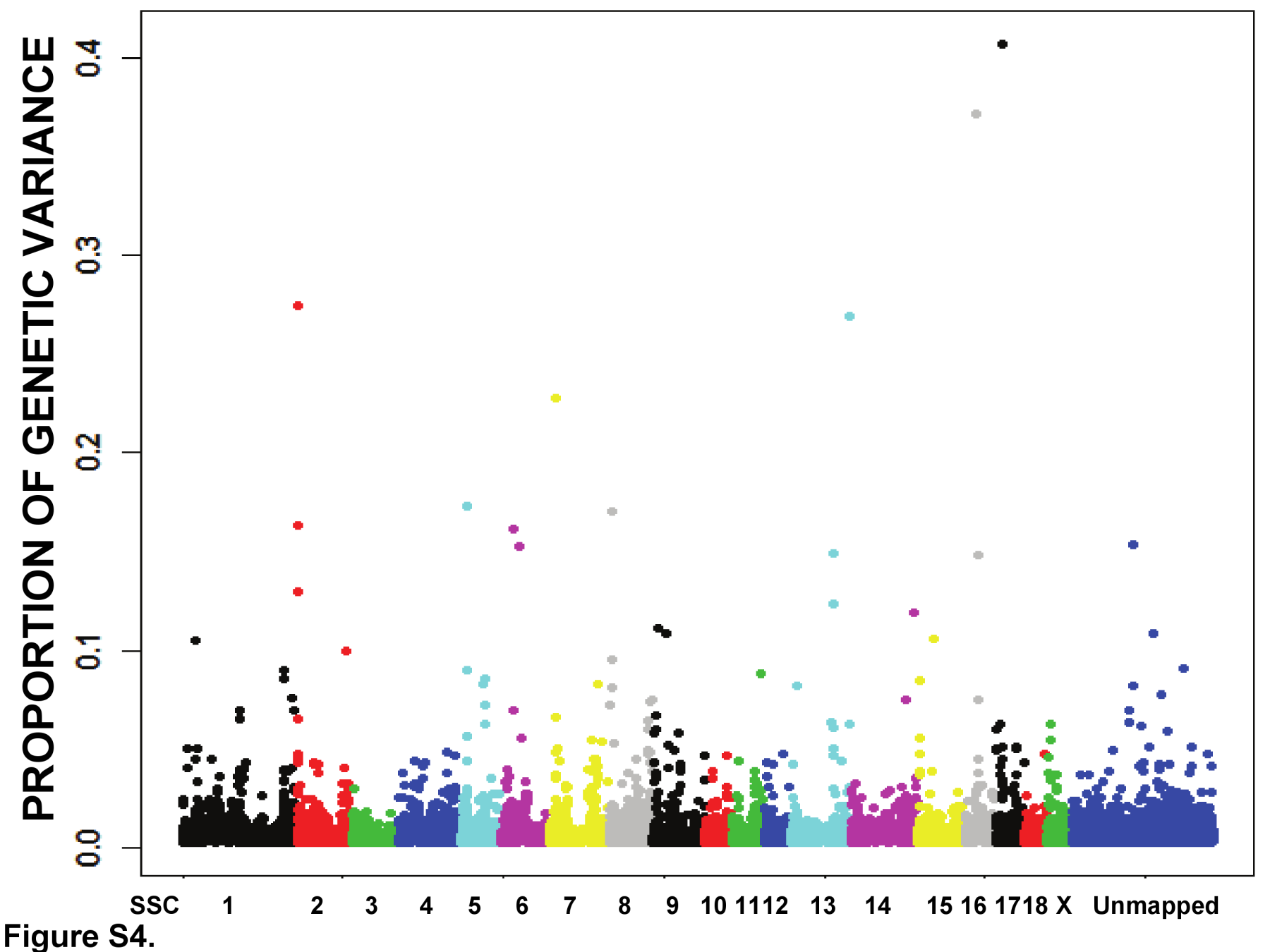

Supplement: Figure S4 — The plots of model frequency of SNPs for assessing the associations between the markers and 10th rib loin muscle area. The X-axis is SNP marker position in genome order, and the Y-axis represents model frequency (0.10 was considered as threshold for 10th rib loin muscle area here). Different colors represent SNPs on different chromosomes from SSC1 to X and unmapped markers. (0.10 MB TIF) [file pone.0014726.s004.tif]

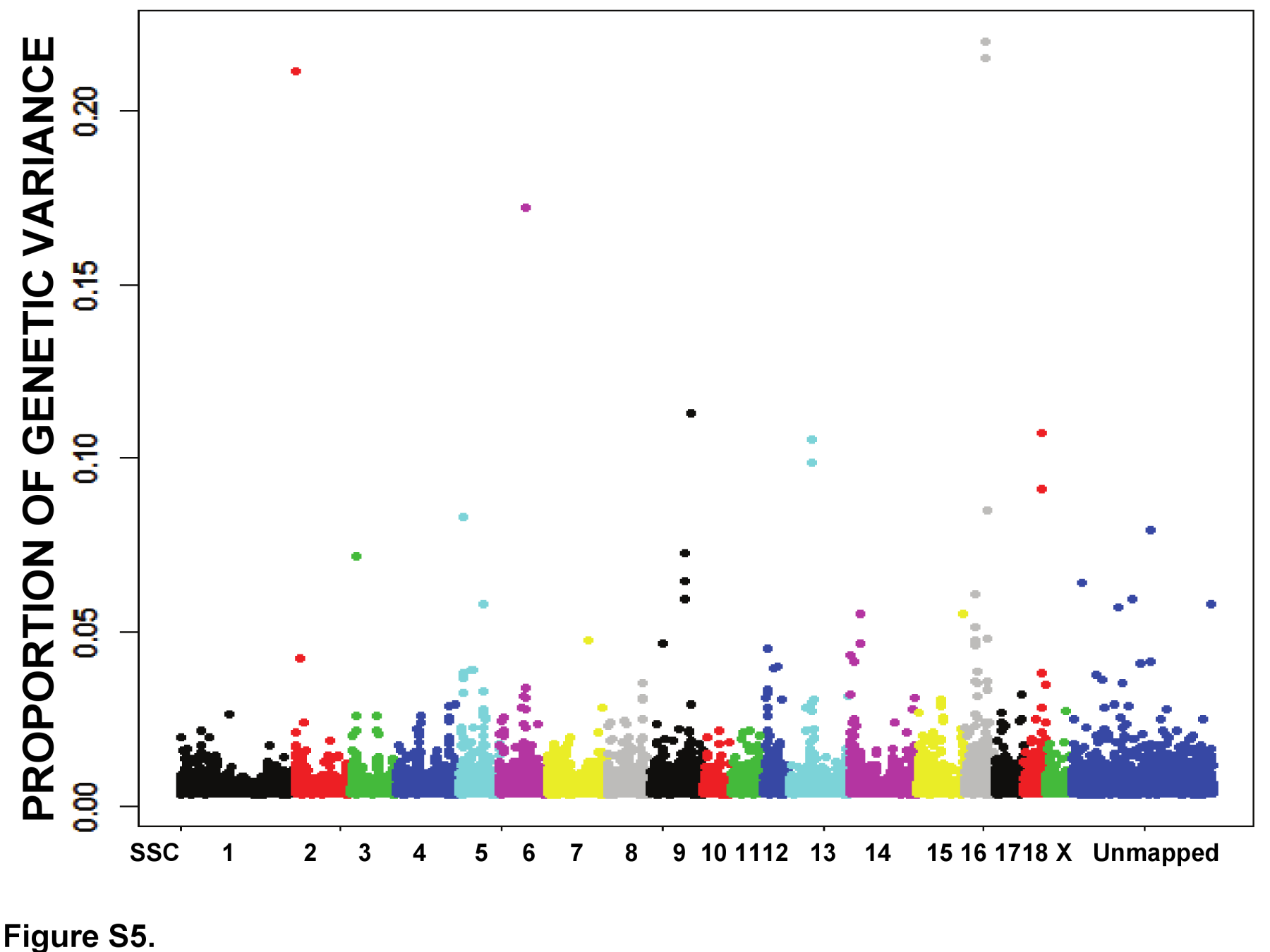

Supplement: Figure S5 — The plots of model frequency of SNPs for assessing the associations between the markers and overall leg action. The X-axis is SNP marker position in genome order, and the Y-axis represents model frequency (0.05 was considered as threshold for overall leg action). Different colors represent SNPs on different chromosomes from SSC1 to X and unmapped markers. (0.10 MB TIF) [file pone.0014726.s005.tif]

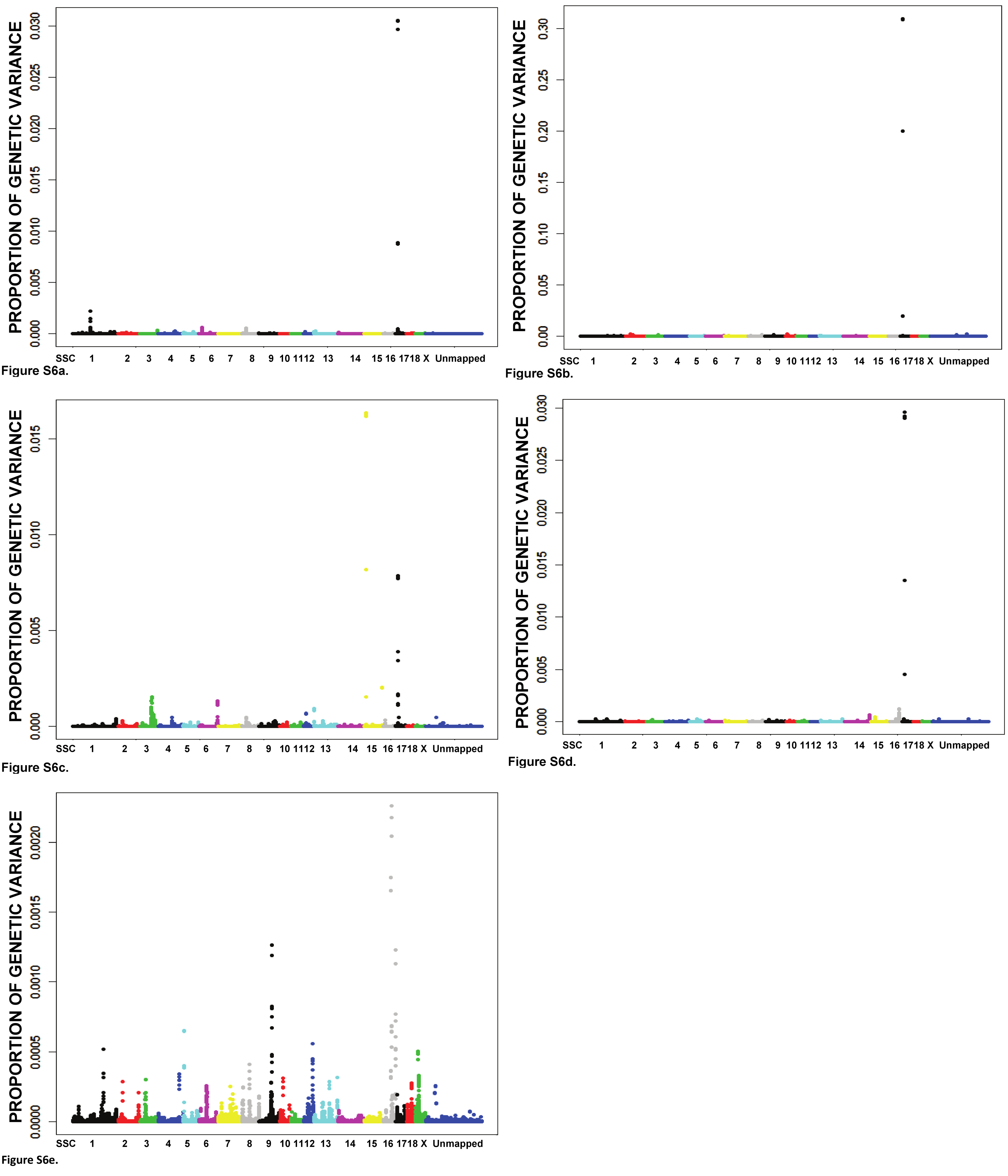

Supplement: Figure S6 — Proportion of genetic variance explained by each window of 5 SNP consecutive markers across the genome for body conformation traits, which was used to determine the candidate genome regions surrounding the significant SNPs. The body conformation traits are a) body length; b) body depth; c) body width; d) rib shape and e) Hip structure. The X-axis is SNP marker position in genome order, and the Y-axis represents accumulative genetic variance of 5-SNP window (the exact candidate regions, the most promising SNPs and P values are shown in Table S7). Different colors represent SNPs on different chromosomes from SSC1 to X and unmapped markers. (1.65 MB TIF) [file pone.0014726.s006.tif]

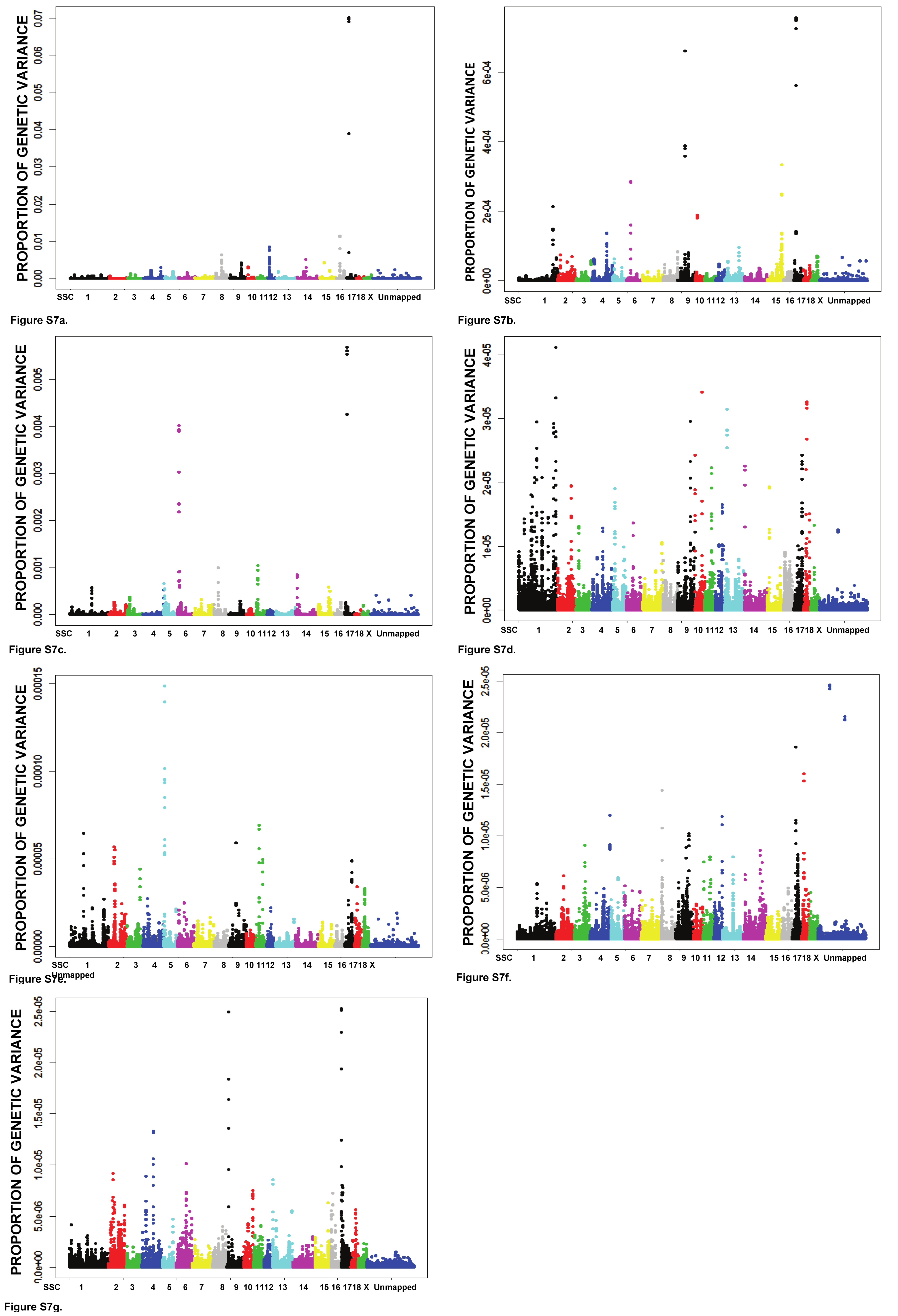

Supplement: Figure S7 — Proportion of genetic variance explained by each window of 5 consecutive SNP markers across the genome for feet and leg structure soundness traits, which was used to determine the candidate genome regions surrounding the significant SNPs. The feet and leg structure soundness traits are a) front leg pastern; b) rear leg pastern; c) front buck knee; d) front feet size; e) rear feet size; f) front uneven toes and g) rear uneven toes. The X-axis is SNP marker position in genome order, and the Y-axis represents accumulative genetic variance of 5-SNP window (the exact candidate regions, the most promising SNPs and P values are shown in Table S9). Different colors represent SNPs on different chromosomes from SSC1 to X and unmapped markers. (2.15 MB TIF) [file pone.0014726.s007.tif]
