# Supplementary material for: Genome-Wide Association Study Identifies Loci for Body Composition and Structural Soundness Traits in Pigs
Source: PLoS One. 2011 Feb 24;6(2):e14726. doi: 10.1371/journal.pone.0014726 (PMC3044704; doi:10.1371/journal.pone.0014726)
Supplement: Table S1 — The description of the 17 analyzed traits of body conformation, feet and leg structure and overall leg action. (0.04 MB DOC) [file pone.0014726.s008.doc]

**Table S1**

| **Trait** | **Description** | **Score 1** | | **Score 9** |
| --- | --- | --- | --- | --- |
| Body length | Distance from tail to scapulae viewed from side | Short | Long | |
| Body depth | Distance from back to sternum viewed from side | Deep | Shallow | |
| Body width | Rump width (Butterfly shape) viewed from rear | Narrow | Wide | |
| Top Line | Arch straightness between shoulder and rump viewed from side | Weak | High topped | |
| Hip structure | Hip line and tail setting viewed from side | Level | Steep | |
| Rib shape | Breast width view from the horizontal | More shape | Less shape | |
| Front turned in/out | Front hocks turned inward/outward from each other viewed from front | Turned out | Turned in | |
| Front pastern posture | Angle of front foot viewed from side | Weak and soft | Upright | |
| Buck knee | Over at the knee of front legs viewed from side | Upright | Severe buck knees | |
| Front foot size | Front foot size | Large | Small | |
| Front uneven toes | Even and uniform shape of front hooves | Even | Severely uneven | |
| Rear turned in/out | Rear hocks turned inward/outward from each other viewed from rear | Turned out | Turned in | |
| Rear pastern posture | Angle of rear foot viewed from side | Weak and soft | Upright | |
| Upright/Weak rear legs | Angle of rear hocks viewed from side | Weak | Upright | |
| Rear foot size | Rear foot size | Large | Small | |
| Rear uneven toes | Even and uniform shape of rear hooves | Even | Severely uneven | |
| Overall leg action | Structural soundness and movement and freedom of other defects | Excellent movement | Most severe/unable to walk | |
