# Supplementary material for: Genome-Wide Association Study Identifies Loci for Body Composition and Structural Soundness Traits in Pigs
Source: PLoS One. 2011 Feb 24;6(2):e14726. doi: 10.1371/journal.pone.0014726 (PMC3044704; doi:10.1371/journal.pone.0014726)
Supplement: Table S2 — The overall statistics about population means, estimated heritability and the genetic parameters of Bayes C analyses for the traits. (0.05 MB DOC) [file pone.0014726.s009.doc]

**Table S2**

| **Trait (Unit)** | **Mean (SD)** | **Posterior mean of Genetic variance** | **Posterior mean of Residual variance** | **Proportion of Variance** | **Estimated heritability** |
| --- | --- | --- | --- | --- | --- |
| 10th rib backfat (cm) | 1.38 (0.36) | 0.061 | 0.052 | 0.541 | 0.65 |
| Last rib backfat (cm) | 1.23 (0.32) | 0.047 | 0.031 | 0.605 | 0.72 |
| Loin muscle area (cm^2^) | 50.82 (5.97) | 15.966 | 13.076 | 0.549 | 0.60 |
| Overall leg action | 4.78 (2.28) | 1.453 | 3.482 | 0.294 | 0.12 |
| Body length | 4.79 (1.01) | 0.178 | 0.631 | 0.211 | 0.29 |
| Body depth | 4.16 (0.48) | 0.312 | 0.784 | 0.284 | 0.34 |
| Body width | 5.39 (1.26) | 0.291 | 0.928 | 0.238 | 0.25 |
| Rib shape | 4.31 (1.58) | 0.359 | 1.876 | 0.161 | 0.26 |
| Hip structure | 4.29 (1.77) | 0.372 | 1.610 | 0.187 | 0.18 |
| Weak top line | 1.18 (0.48) | 0.005 | 0.204 | 0.024 | 0.11 |
| High top line | 1.34 (0.54) | 0.018 | 0.269 | 0.065 | 0.12 |
| Front leg pastern posture | 4.59 (1.69) | 0.898 | 1.809 | 0.331 | 0.28 |
| Front leg buck knees | 4.68 (1.71) | 0.463 | 2.135 | 0.178 | 0.13 |
| Front legs turned in | 2.07 (0.70) | 0.023 | 0.456 | 0.048 | 0.02 |
| Front feet size | 5.25 (0.95) | 0.071 | 0.676 | 0.095 | 0.16 |
| Uneven front toes | 2.19 (0.99) | 0.061 | 0.867 | 0.066 | 0.09 |
| Rear leg pastern posture | 4.28 (1.40) | 0.229 | 1.569 | 0.127 | 0.31 |
| Weak rear legs | 1.56 (0.81) | 0.059 | 0.603 | 0.089 | 0.14 |
| Upright rear legs | 1.43 (0.73) | 0.068 | 0.455 | 0.130 | 0.21 |
| Rear legs turned in | 2.03 (0.38) | 0.110 | 0.457 | 0.194 | 0.14 |
| Rear feet size | 5.18 (1.01) | 0.097 | 0.759 | 0.114 | 0.13 |
| Uneven rear toes | 2.31 (1.09) | 0.059 | 1.062 | 0.053 | 0.12 |

*The estimated heritability of the analyzed traits are from Nikkilä et al. (2008). Posterior mean of genetic variance, residual variance and proportion of variance accounted for by SNPs were derived from Bayes C analyses.

**Overall leg action, body conformation and feet and leg structural soundness were scored with a 9-point scale.

*** Four traits with intermediate optimum were divided into two traits. Top line: weak top line and high top line; Turned front leg: front legs turned in and front legs turned out; Turned rear leg: rear legs turned in and rear legs turned out; Upright/weak rear legs: upright rear legs and weak rear legs. The estimates of front legs turned out and rear legs turned out are not shown because of small size of animals.
