# Supplementary material for: Genome-Wide Association Study Identifies Loci for Body Composition and Structural Soundness Traits in Pigs
Source: PLoS One. 2011 Feb 24;6(2):e14726. doi: 10.1371/journal.pone.0014726 (PMC3044704; doi:10.1371/journal.pone.0014726)
Supplement: Table S3 — The overall statistics on average interval between SNPs and SNP distribution of porcine 60K SNP array. (0.05 MB DOC) [file pone.0014726.s010.doc]

**Table S3**

| **SSC** | **SNP no.** | **Physical size (Mb)** | | **Mb/SNP** | | **Linkage map (cM)** | | **cM/SNP** | **Intra-genic SNP no.** | | | **Gene no.** | | **Max. SNP no.** |
| --- | --- | --- | --- | --- | --- | --- | --- | --- | --- | --- | --- | --- | --- | --- |
| 1 | 7139 | 295 | 0.041 | | 144 | | 0.020 | | | 1911 | 832 | | 33 | |
| 2 | 3482 | 140 | 0.040 | | 132 | | 0.038 | | | 1058 | 516 | | 20 | |
| 3 | 2880 | 123 | 0.043 | | 129 | | 0.045 | | | 819 | 402 | | 15 | |
| 4 | 3839 | 136 | 0.035 | | 130 | | 0.034 | | | 1140 | 520 | | 18 | |
| 5 | 2520 | 100 | 0.039 | | 114 | | 0.045 | | | 747 | 379 | | 18 | |
| 6 | 3011 | 123 | 0.041 | | 165 | | 0.055 | | | 928 | 455 | | 12 | |
| 7 | 3700 | 136 | 0.037 | | 156 | | 0.042 | | | 1192 | 576 | | 22 | |
| 8 | 2717 | 120 | 0.044 | | 127 | | 0.047 | | | 550 | 263 | | 17 | |
| 9 | 3269 | 132 | 0.040 | | 138 | | 0.042 | | | 876 | 381 | | 25 | |
| 10 | 1722 | 67 | 0.039 | | 124 | | 0.072 | | | 438 | 166 | | 23 | |
| 11 | 2021 | 80 | 0.039 | | 85 | | 0.042 | | | 366 | 150 | | 14 | |
| 12 | 1583 | 57 | 0.036 | | 113 | | 0.071 | | | 612 | 318 | | 14 | |
| 13 | 3745 | 145 | 0.039 | | 126 | | 0.034 | | | 867 | 425 | | 18 | |
| 14 | 4203 | 148 | 0.035 | | 111 | | 0.026 | | | 1477 | 644 | | 28 | |
| 15 | 2962 | 134 | 0.045 | | 112 | | 0.038 | | | 672 | 306 | | 16 | |
| 16 | 1932 | 77 | 0.039 | | 93 | | 0.048 | | | 399 | 158 | | 12 | |
| 17 | 1793 | 64 | 0.036 | | 97 | | 0.054 | | | 532 | 255 | | 20 | |
| 18 | 1361 | 54 | 0.039 | | 57 | | 0.042 | | | 426 | 157 | | 16 | |
| X | 1548 | 126 | 0.081 | | 128 | | 0.083 | | | 378 | 196 | | 13 | |
| Y | 19 | - | - | | - | | - | | | - | - | | - | |
| Unmapped | 8786 | - | - | | - | | - | | | - | - | | - | |
| Summary | 64232 | 2257 | - | | 2281 | | - | | | 15388 | 7099 | | - | |

^*^ The physical size is based on *Sus scrofa* Build 9 (http://www.ensembl.org/Sus_scrofa/Info/Index), and the linkage map is based on USDA-MARC v2 (A) (http://www.thearkdb.org/). SNP distribution search was performed using *Sus Scrofa* Build 9.
