# Supplementary material for: Genome-Wide Association Study Identifies Loci for Body Composition and Structural Soundness Traits in Pigs
Source: PLoS One. 2011 Feb 24;6(2):e14726. doi: 10.1371/journal.pone.0014726 (PMC3044704; doi:10.1371/journal.pone.0014726)
Supplement: Table S4 — The detail information about candidate regions and the most significant SNPs associated with last rib backfat. (0.05 MB DOC) [file pone.0014726.s011.doc]

**Table S4**

| **SSC** | **Location (Start-End, Mb)** | **Most significant SNP** | **Genes** | **P value** |
| --- | --- | --- | --- | --- |
| 1 | 165.65-168.84 | INRA0004898 ALGA0006599 ALGA0006623 ASGA0005017 | *VPS4 KDSR SERPINB5 BCL2***^*^** *PHLPP1 ZCCHC2 TNFRSF11A KIAA1468 RNP152 CDH20 MC4R***^*^** *TN3 CCBE1***^*^** *LMAN1 CPLX4 RAX SEC11C* | < 0.001 |
| 1 | 223.02-224.41 | INRA0005938 ALGA0007864 | *C9orf123* | < 0.001 |
| 2 | 1.14-1.46 | ALGA0103099 | *SHANK2 FADD PPFIA1 CTTN* | < 0.01 |
| 2 | 6.26-6.47 | H3GA0005813 | *C11orf20 ESRRA TRMT112 PRDX5 KCNK4 GPR137 BAD PLCB3 PPP1R14B FKBP2 VEGFB DNAJC4 NUDT22 TRPT1 FERMT3 CCDC88B MACROD1 FLRT1 OTUB1 NAT11 C11orf84* | < 0.01 |
| 4 | 9.39-9.62 | M1GA0005682 | *ADCY8 ASAP1 FAM49B* | < 0.01 |
| 4 | 79.33-80.12 | M1GA0005986 | *XKR4 RP1 TCEA1P2 RGS20 ATP6V1H***^*^** *OPRK1***^*^** *ST18* | < 0.001 |
| 5 | 63.55-64.75 | MARC0059561 ASGA0026127 H3GA0016710 | *PLA2G6***^*^** *BAIAP2L2 SLC16A8 PICK1 SOX10 POLR2F C22orf23 MICALL1 EIF3L* | < 0.01 |
| 6 | 0.64 | ASGA0084674 | *-* | < 0.05 |
| 6 | 8.79 | H3GA0017561 | *TERF2IP KARS ADAT1 GABARAPL2 TMEM170 CFDP1 LDHD***^*^** | < 0.001 |
| 7 | 10.60-11.04 | ASGA0031167 ALGA0038566 | *GFOD1 S IRT5 RANBP9 CCDC90A RNF182 CD83* | < 0.01 |
| 8 | 15.13-15.53 | ALGA0046784 | *DHX15 ANAPC4 SLC34A2* | < 0.001 |
| 9 | 43.67-43.93 | ASGA0094905 | *RNF214 BACE1 CEP164 DSCAML1 FXYD2* | < 0.01 |
| 13 | 52.64-53.01 | ALGA0070637 | *-* | < 0.05 |
| 14 | 135.96-136.21 | ASGA0067120 | *BAG3 C10orf119 SEC23IP PPAPDC1A BRWD2* | < 0.01 |
| 14 | 139.09-139.38 | M1GA0019394 | *PSTK IKZF5 ACADSB***^*^** *HMX3 HMX2 BUB3 GPR26 CPXM2* | < 0.05 |
| 15 | 129.67-130.02 | MARC0057052 | *COL6A3 RAB17 RAMP1 ESPNL FAM132B PER2 ASB1* | < 0.05 |
| 16 | 29.45-30.00 | H3GA0046417 | *PARP8 ISL1***^*^**  *PELO ITGA1 UBL5 MOCS2 FST NDUFS4 ARL15* | < 0.01 |
| 17 | 44.02-44.53 | ALGA0095204 | *SLC32A1 ACTR5 PPP1R16B FAM83D DHX35 TOP1 PLCG1* | < 0.01 |
| 17 | 46.69-47.79 | ASGA0090592 ALGA0095340 | *EMILIN3 LIPIN3 CHD6 SFRS6* | < 0.01 |
| 18 | 14.15-14.78 | ALGA0118164 | *CHCHD3***^*^**  *PLXNA4* | < 0.05 |
| X | 122.23-122.71 | ASGA0081603 | *ZNF707 ATP2B3***^*^**  *FAM58A BCAP31 PNCK SRPK3 SSR4 PDZD4 L1CAM V2R ARHGAP4 ARD1A* | < 0.01 |

^*^ The genes labeled with superscript star sign indicated are those potentially important ones relevant to fat metabolism using functional annotation through online DAVID (http://david.abcc.ncifcrf.gov/). P values indicated the significant candidate regions and were determined from bootstrap analysis based on the genetic variance of 5-SNPs sliding window.
