# Supplementary material for: Genome-Wide Association Study Identifies Loci for Body Composition and Structural Soundness Traits in Pigs
Source: PLoS One. 2011 Feb 24;6(2):e14726. doi: 10.1371/journal.pone.0014726 (PMC3044704; doi:10.1371/journal.pone.0014726)
Supplement: Table S5 — The detail information about the putative candidate regions and the most significant SNPs associated with 10th rib backfat. (0.05 MB DOC) [file pone.0014726.s012.doc]

**Table S5.**

| **SSC** | **Location (Start-End, Mb)** | **Most significant SNP** | **Gene** | **P value** |
| --- | --- | --- | --- | --- |
| 1^*^ | 31.11-31.55 | ALGA0002231 | *SLC2A12 TBP1L TCF21***^*^** *EYA4* | < 0.05 |
| 1^*^ | 165.47-168.63 | INRA0004898 ALGA0006599 ALGA0006623 ASGA0005017 | *VPS4 KDSR SERPINB5 BCL2***^*^** *PHLPP1 ZCCHC2 TNFRSF11A KIAA1468 RNP152 CDH20 MC4R***^*^** *TN3 CCBE1***^*^** *LMAN1 CPLX4 RAX SEC11C* | < 0.1 |
| 2^*^ | 18.71-19.50 | MARC0039631 | *-* | < 0.05 |
| 2 | 120.31-120.38 | MARC0019720 | *KIAA1024L* | < 0.05 |
| 2 | 125.94-126.21 | ASGA0104950 | *TRPC7 SMAD5***^*^** *SPOCK1* | < 0.05 |
| 4^*^ | 8.29-8.94 | ASGA0018107 ASGA0018114 | *ADCY8* | < 0.05 |
| 4^*^ | 79.33-80.12 | M1GA0005986 | *XKR4 RP1 TCEA1P2 RGS20 ATP6V1H***^*^** *OPRK1***^*^** | < 0.05 |
| 6^*^ | 8.69-8.94 | H3GA0017561 | *TERF2IP KARS GABARAPL2 TMEM170 CFDP1 LDHD* | < 0.1 |
| 6^*^ | 50.71-51.08 | H3GA0052889 | *FHAD1 EFHD2***^*^** | < 0.05 |
| 7^*^ | 103.61-104.06 | M1GA0010637 | *SIPA1L1 DPF3* | < 0.1 |
| 7 | 123.46-123.95 | M1GA0010841 | *PRIMA1 ASB2 FAM181A OTUB2 DDX24 ISG12 CBG A1AT* | < 0.05 |
| 11 | 77.93-78.35 | H3GA0032842 H3GA0032852 | *ARHGEF7 TUBGCP3 GRTP1* | < 0.05 |
| 12 | 38.48-38.80 | MARC0085508 | *TRIM65 MRPL38 FBF1 ACOX1***^*^** *TRIM47* | < 0.05 |
| 16 | 2.59-2.83 | ASGA0071923 | *-* | < 0.05 |
| 16 | 14.83-15.09 | ASGA0072382 | *-* | < 0.1 |
| 18^*^ | 14.15-14.78 | ALGA0118164 | *CHCHD3***^*^** *PLXNA4* | < 0.1 |
| 18 | 23.37-23.63 | ASGA0079343 | *AASS PTPRZ1 FAM3C WNT16***^*^** | < 0.1 |
| X | 122.1-122.6 | ASGA0081603 | *NSDHL***^*^** *ZNF185 ZNF707 ATP2B3***^*^** *FAM58A BCAP31 PNCK* | < 0.05 |

^*^ The chromosomes labeled with superscript asterisk indicate the regions corresponding to the reported QTL associated with 10th rib backfat (http://www.animalgenome.org/cgi-bin/QTLdb/SS/index). The genes labeled with superscript asterisk indicate those potentially important ones relevant to fat metabolism using functional annotation through online DAVID (http://david.abcc.ncifcrf.gov/). P values indicate the significance of candidate regions, which were determined from the bootstrap analysis of genetic variance of sliding window of 5-SNPs.
