# Supplementary material for: Genome-Wide Association Study Identifies Loci for Body Composition and Structural Soundness Traits in Pigs
Source: PLoS One. 2011 Feb 24;6(2):e14726. doi: 10.1371/journal.pone.0014726 (PMC3044704; doi:10.1371/journal.pone.0014726)
Supplement: Table S6 — The detail information about the putative candidate regions and the most significant SNPs associated with 10th rib loin muscle area. (0.05 MB DOC) [file pone.0014726.s013.doc]

**Table S6**

| **SSC** | | **Location (Start-End, Mb)** | | **Most significant SNP** | **Gene** | **P value** |
| --- | --- | --- | --- | --- | --- | --- |
| 1^*^ | 24.30-24.40 | | ASGA0001646 | | *VTA1 NMBR* | ns |
| 2^*^ | 0.81-1.26 | | MARC0022036 | | *OSBPL5 SHANK2* | < 0.05 |
| 2^*^ | 2.15-2.67 | | M1GA0002180 M1GA0002244 | | *IGF2***^*^** *TPCN2 MRGRRF MRGPRD IGHMBP2***^*^** *MRPL21 CPT1 MTL5 GALA SAPS3* | < 0.001 |
| 5 | 14.53-14.91 | | M1GA0007663 | | *KIAA1602 AQP2 AQP5***^*^** *AQP6 SMARCD1 ACCN3 RACGAP1P LARP4* | < 0.01 |
| 6^*^ | 23.57-23.77 | | ASGA0093489 | | *SALL1 CYLD ZNF423* | < 0.01 |
| 6^*^ | 47.67-48.15 | | M1GA0008601 | | *PEX14***^*^** *TARDBP MASP2 SRM EXOSC10 ANGPTL7* | < 0.05 |
| 7^*^ | 14.39-14.63 | | ASGA0094630 | | *TPMT AOF1 DEK RNF144B* | < 0.01 |
| 8 | 8.76-8.97 | | H3GA0052944 | | *-* | < 0.01 |
| 9 | 9.87-10.10 | | ALGA0051272 | | *DGAT2 WNT11***^*^** *PRKRIR* | ns |
| 9^*^ | 27.97-28.11 | | ASGA0042377 | | *-* | ns |
| 13 | 99.79-100.65 | | ALGA0072022 H3GA0037306 | | *DLG1***^*^**  *SENP5 MFI2 PAK2***^*^** *PCYT1A OSTA* | < 0.001 |
| 13^*^ | 142.91-143.13 | | ASGA0060118 | | *ETS2***^*^** *B3GALT5* | < 0.05 |
| 14 | 141.91-142.13 | | MARC0062974 | | *C10orf122 C10orf137 UROS BCCIP DHX32* | < 0.05 |
| 15^*^ | 42.76-43.00 | | MARC0084849 | | *IRF2***^*^**  *CASP3***^*^** *MLF1IP CCDC111* | < 0.05 |
| 16 | 27.82-27.92 | | INRA0051405 | | *ISL1***^*^** *RARP8 PELO ITGA1***^*^** *ITGA2***^*^**  *UBL5* | < 0.1 |
| 16 | 30.42-30.67 | | ALGA0090171 | | *MOCS2 FST***^*^** *NDUFS4***^*^** *ARL15* | < 0.05 |
| 17^*^ | 15.24-15.91 | | INRA0052808 | | *BMP2***^*^** | < 0.001 |

^*^ The chromosomes labeled with superscript asterisk indicate the regions corresponding to the reported QTL associated with 10th loin muscle area (http://www.animalgenome.org/cgi-bin/QTLdb/SS/index). The genes labeled with superscript asterisk indicate those potentially important ones relevant to muscle development using functional annotation through online DAVID (http://david.abcc.ncifcrf.gov/). P values indicate the significant confidence of candidate regions, which were determined from the genetic variance of 5-SNPs sliding window obtained by bootstrap analysis.
