# Supplementary material for: Genome-Wide Association Study Identifies Loci for Body Composition and Structural Soundness Traits in Pigs
Source: PLoS One. 2011 Feb 24;6(2):e14726. doi: 10.1371/journal.pone.0014726 (PMC3044704; doi:10.1371/journal.pone.0014726)
Supplement: Table S7 — The detail information about candidate regions and the most significant SNPs associated with body conformation traits. (0.09 MB DOC) [file pone.0014726.s014.doc]

**Table S7**

| **Trait** | **SSC** | **Location  (Start-End, Mb)** | **Most significant SNP** | **Genes** | **P value** |
| --- | --- | --- | --- | --- | --- |
| body length | 1 | 98.61-98.94 | ALGA0005041 | *SETBP1 SLC14A2 SLC14A1* | < 0.001 |
|  | 1 | 267.77-271.63 | ASGA0104765 | *COL27A1***^*^** *AGP AKNA DFNB31 C9orf91 TENA TNFRSF8 PAPPA***^*^** *TRIM32 ASTN2 TLR4* | < 0.01 |
|  | 3 | 117.87-118.64 | H3GA0011038 MARC0022891 | *RNF144A CMPK2***^*^**  *RSAD2* | < 0.01 |
|  | 6 | 15.41-16.09 | ALGA0034888 H3GA0017700 | *CDH11* | < 0.01 |
|  | 17 | 14.95-15.45 | MARC0070553 INRA0052808 | *BMP2***^*^** | < 0.001 |
|  | 17 | 17.25-17.47 | H3GA0047994 | *PLCB1***^*^** | < 0.001 |
|  | 17 | 18.50-18.57 | ASGA0075659 | *PLCB4***^*^** *PAK7 ANKRD5***^*^** | < 0.01 |
| body depth | 1 | 270.15-270.26 | ALGA0009685 | *PAPPA***^*^** *TRIM32* | < 0.001 |
|  | 8 | 103.48-103.65 | ASGA0091053 | *MTP C4orf17 ADH7 ADH5 ADH4 METAP1* | < 0.001 |
|  | 8 | 104.25-104.32 | ALGA0049484 | *C4orf37* | < 0.001 |
|  | 10 | 6.11-6.18 | ASGA0046081 | *-* | < 0.001 |
|  | 10 | 10.23-10.34 | ASGA0046469 | *MOSC1 HLX* | < 0.001 |
|  | 17 | 14.95-15.45 | MARC0070553 INRA0052808 | *BMP2***^*^** | < 0.001 |
| body width | 3 | 77.28-80.14 | H3GA0010000 MARC0089608 ALGA0111992 | *FANCL VRK2 CCDC85A EFEMP1 PNPT1 CCDC104 C2orf63 UBIQ MTIF2 CCDC88A RTN4* | < 0.01 |
|  | 6 | 116.23-117.53 | ASGA0098091 | *SPATA6 SLC5A9* | < 0.01 |
|  | 13 | 2.71-2.84 | ALGA0067532 | *GALNTL2 DPH3 OXNAD1***^*^** *RFTN1* | < 0.01 |
|  | 15 | 7.27-7.45 | ALGA0083701 ASGA0068496 | *LYPD6* | < 0.001 |
|  | 15 | 126.66-16.88 | ALGA0088210 | *ASB18 GBX2***^*^** *IQCA1 CXCR7* | < 0.001 |
|  | 17 | 13.45-14.50 | ASGA0075536 | *RASSF2 C20orf196 SCG1 TRMT6 MCM8 CRLS1 LRRN4* | < 0.001 |
|  | 17 | 14.90-15.45 | MARC0074172 MARC0070553 INRA0052808 | *BMP2***^*^** | < 0.001 |
|  | 17 | 19.39-19.64 | H3GA0048042 | *ANKRD5***^*^** *MKKS***^*^** *RPLP1 C20orf94 JAG1* | < 0.001 |
| Rib shape | 13 | 136.23-136.40 | ALGA0073505 | *ADAMTS5 C21orf7* | < 0.01 |
|  | 16 | 58.32-58.99 | ALGA0091126 | *CCNG1 GABRG2 GABRA1* | < 0.001 |
|  | 17 | 11.52 | ASGA0075278 | *-* | < 0.001 |
|  | 17 | 15.38-15.45 | MARC0070553 INRA0052808 | *BMP2***^*^** | < 0.001 |
| Hip structure | 2 | 23.8-23.9 | H3GA0006388 | *LDLRAD3***^*^** *TRIM44 SLC1A2* | < 0.01 |
|  | 4 | 118.61-119.13 | ALGA0028437 | *AMYP* | < 0.01 |
|  | 5 | 2.96-3.37 | H3GA0015260 | *CP2DP TCF20***^*^** *NFAM1* | < 0.01 |
|  | 8 | 59.42-59.84 | MARC0103006 | *PLF4 PPBP***^*^** *AMC2 CXCL2 MTHFD2l***^*^** *EREG AREG EPGN* | < 0.001 |
|  | 9 | 72.27-73.03 | ALGA0053916 ALGA0053920 ALGA0053925 H3GA0027825 ALGA0053928 ASGA0043847 ASGA0043850 | *ASNS C1GALT1 COL28A1***^*^** | < 0.01 |
|  | 13 | 142.90-143.95 | ASGA0060105 M1GA0017861 | *KCNJ15 ETS2***^*^** *B3GALT5 IGSF5 DSCAM BACE2 MX2 MX1* | < 0.01 |
|  | 16 | 75.30-76.57 | MARC0032852 ASGA0099242  H3GA0047519 M1GA0021543 | *IRX4 MRPL36 NDUFS6* | < 0.01 |
|  | 18 | 29.36-29.84 | MARC0088381 | *-* | < 0.001 |
|  | 18 | 41.78 | ALGA0098337 | *PRR15***^*^** *CHN2 CPVL* | < 0.001 |
|  | X | 13.12-13.94 | INRA0056524 | *REPS2 NHS SCML1 RAI2***^*^** *BEND2 SCML2* | < 0.001 |
| Weak top line | 4 | 106.61 | ASGA0021489 | *TBX15 SPAG17 WDR3 GDAP2* | < 0.01 |
|  | 13 | 139.28-139.33 | H3GA0037912 H3GA0037916  ALGA0073715 | *ATP5O***^*^** *MRPS6* | < 0.01 |
| High top line | 4 | 128.62 | ALGA0029201 | *-* | < 0.01 |
|  | 6 | 116.10-116.12 | ALGA0106508 ALGA0122941 MARC0068985 | *BEND5 SPATA6* | < 0.001 |
|  | 13 | 7.02 | ALGA0067800 | *ZNF385D* | < 0.001 |
|  | 17 | 15.38 | MARC0070553 | *BMP2***^*^** *PLCB1***^*^** | < 0.01 |
|  | 17 | 17.48 | H3GA0047994 | *PLCB4***^*^** | < 0.01 |

^*^ The genes labeled with a superscript asterisk indicate those potentially important ones relevant to skeleton development, bone and cartilage development, and energy metabolism using functional annotation through online DAVID (http://david.abcc.ncifcrf.gov/). P values indicate the significant candidate regions and determined from bootstrap analysis based on the genetic variance of 5-SNPs sliding windows.
