# Supplementary material for: Genome-Wide Association Study Identifies Loci for Body Composition and Structural Soundness Traits in Pigs
Source: PLoS One. 2011 Feb 24;6(2):e14726. doi: 10.1371/journal.pone.0014726 (PMC3044704; doi:10.1371/journal.pone.0014726)
Supplement: Table S8 — The detail information about the putative candidate regions and the most significant SNPs associated with overall leg action. (0.05 MB DOC) [file pone.0014726.s015.doc]

**Table S8**

| **SSC** | **Location (Start-End, Mb)** | **Most significant SNP** | **Gene** | **P value** |
| --- | --- | --- | --- | --- |
| 2 | 0.81-1.26 | MARC0022036 | *NADSYN1 OSBPL5 SHANK2* | *< 0.001* |
| 3 | 11.83-11.99 | MARC0056315 | *-* | *< 0.01* |
| 5 | 10.77-10.94 | DIAS0003292 | *FBXO7 BPIL2 C22orf28 ASCL4 PRDM4***^*^** *PWP1 BTBD11* | *< 0.01* |
| 5 | 61.97 | MARC0036560 | *CCND2***^*^** *PARP11* | *< 0.01* |
| 6 | 63.29-63.83 | ASGA0028870 | *GRIK3 MTF1 SF3A3 FHL3***^*^**  *UTP11L POU3F1* | *< 0.001* |
| 9 | 81.84-83.28 | H3GA0027878 ALGA0054178 ALGA0054186 | *HDAC9 TWIST1***^*^**  *FERD3L TMEM196 SP4***^*^** | *< 0.01* |
| 9 | 101.51-102.27 | ALGA0054561 | *EPDR1***^*^** *ZNF786 ERP72 CUL1* | *< 0.01* |
| 13 | 48.45-48.89 | ALGA0070448 ASGA0057807 | *CHL1 CNTN6 CNTN4***^*^** | *< 0.01* |
| 14 | 27.77 | ALGA0076305 | *RIMBP2 PIWIL1 FZD10***^*^** | *ns* |
| 15 | 131.44-133.13 | ALGA0102768 | *GPC1 ANKMY1 DUSP28 RNPEPL1 FARP2 STK25 BOK THAP4 PPP1R7 MTERFD2 SNED1* | *< 0.01* |
| 16 | 23.6-24.5 | ALGA0106657 ALGA0110358 | *FBXO4 GHR***^*^** *CCDC15 CCL28 NNT PLCXD3 C7* | *< 0.001* |
| 16 | 26.13-26.47 | ALGA0089983 H3GA0046376 | *FGF10***^*^** *MRPS30* | *< 0.001* |
| 16 | 55.28-55.81 | H3GA0046827 H3GA0046828 | *-* | *< 0.001* |
| 16 | 58.65-59.61 | H3GA0046858 | *GABRA1 GABRG2 GABRB2 ATP10B* | *< 0.01* |
| 18 | 44.49-44.88 | H3GA0051007 MARC0033103 | *HOXA3***^*^**  *HOXA1***^*^** *HOXA2***^*^** *SKAP2 SNX10 CBX3* | *< 0.001* |

^*^ The genes labeled with superscript star sign indicated are those potentially important ones relevant to skeleton development, bone and muscle development and growth using functional annotation through online DAVID (http://david.abcc.ncifcrf.gov/). P values indicated the significant confidence of candidate regions, which were determined the accumulative genetic variance of 5-SNPs sliding window.
