# Supplementary material for: Genome-Wide Association Study Identifies Loci for Body Composition and Structural Soundness Traits in Pigs
Source: PLoS One. 2011 Feb 24;6(2):e14726. doi: 10.1371/journal.pone.0014726 (PMC3044704; doi:10.1371/journal.pone.0014726)
Supplement: Table S9 — The detail information about candidate regions and the most significant SNPs associated with feet and leg structural soundness traits. (0.19 MB DOC) [file pone.0014726.s016.doc]

**Table S9**

| **Trait** | **SSC** | **Location  (Start-End, Mb)** | **Most significant SNP** | **Genes** | **P value** |
| --- | --- | --- | --- | --- | --- |
| Front leg pastern | 3 | 18.82-18.89 | ALGA0017963 | *HS3ST4* | <0.01 |
|  | 4 | 44.28-44.58 | MARC0073869 | *C8orf83* | < 0.01 |
|  | 4 | 111.26-111.52 | ALGA0027847 | *Rsbn1 PHTF1 MAGI3 LIG-2* | <0.01 |
|  | 5 | 61.75-61.97 | H3GA0016584 ALGA0032500 MARC0036560 | *FGF6***^*^** *FGF23***^*^** *CCND2* | < 0.01 |
|  | 8 | 8.06-8.13 | H3GA0056471 | *CC2D2A FBXL5 FGFBP1***^*^** | < 0.05 |
|  | 8 | 58.30-58.86 | ASGA0038910 | *COX18 RASSF6* | < 0.01 |
|  | 8 | 68.27-68.42 | ASGA0039113 | *ATP5I***^*^** | < 0.01 |
|  | 9 | 76.44-76.77 | INRA0032114 | *TMEM106B ARL4A***^*^** *SCIN* | < 0.01 |
|  | 9 | 81.74-82.84 | ALGA0054178 ALGA0054186 | *PRPS1 TWIST1***^*^** *TMEM196 SP4***^*^** | < 0.01 |
|  | 10 | 1.91-2.03 | ALGA0056284 ALGA0056299 | *RGS2* | < 0.001 |
|  | 12 | 6.86-7.93 | H3GA0033370 ALGA0114079 ASGA0053037 | *SOX9***^*^** | < 0.01 |
|  | 13 | 10.52-10.69 | ALGA0068125 | *NGLY1 OXSM* **^*^**  *LRRC3B* | < 0.001 |
|  | 14 | 45.48-45.28 | ALGA0077321 | *CRBB1 CRYBA4 MN1* | < 0.05 |
|  | 14 | 57.01-57.28 | BGIS0004826 | *GNG4 B3GALNT2 TBCE GGPS1 ARID4B RBM34 TOMM20* | < 0.05 |
|  | 15 | 28.28-28.38 | H3GA0044119 | *CRTR1 TFCP2L1 GLI2***^*^** | < 0.001 |
|  | 15 | 80.35-80.49 | ASGA0070136 | *-* | < 0.01 |
|  | 16 | 24.54-24.64 | ALGA0106657 ALGA0110358 | *FBXO4 GHR***^*^**  *CCDC15 CCL28 NNT PLCXD3 C7* | < 0.001 |
|  | 16 | 26.13-26.26 | ALGA0089983 | *FGF10***^*^**  *MRPS30* | < 0.001 |
|  | 16 | 58.65-58.99 | H3GA0046858 | *GABRG2 GABRA1 GABRB2* | < 0.01 |
|  | 17 | 14.95-15.45 | MARC0070553 INRA0052808 | *BMP2***^*^** | < 0.001 |
| Rear leg pastern | 1 | 263.85-263.92 | ASGA0089933 | *PALM2 AKAP2 C9orf152 THIO TXNDC8* | <0.01 |
|  | 1 | 287.64-287.89 | INRA0007890 | *NTNG2 BARHL1 GTF3C4* | < 0.001 |
|  | 3 | 115.78-115.84 | M1GA0024147 | *TRIB2 LPIN1***^*^** *NTSR2***^*^** | < 0.01 |
|  | 4 | 15.56-15.71 | ASGA0018674 | *FBX32 WDYHV1 ATAD2 ZHX1* | < 0.001 |
|  | 6 | 26.64-26.76 | ASGA0100291 ASGA0091288  ALGA0104223 | *UQCRFS1 PLEKHF1 C19orf12 POP4* | < 0.01 |
|  | 9 | 48.55-49.16 | MARC0112820 H3GA0027376 | *C11orf63 BSX***^*^** *GRAMD1B ZNF202 OR6X1 OR6M1* | < 0.001 |
|  | 10 | 11.28-11.32 | H3GA0056488 | *DUSP10***^*^** *HHIPL2 TAF1A* | < 0.01 |
|  | 15 | 120.70-120.97 | ASGA0090255 ALGA0113553  MARC0079431 | *IRS1***^*^** *RHBDD1 COL4A4***^*^** *COL4A3* **^*^** *MFF TM4SF20* | < 0.01 |
|  | 15 | 123.41-123.53 | ASGA0071340 | *DNER***^*^** *FBXO36 SLC16A14 SP110 SP140* | < 0.01 |
|  | 15 | 126.88 | ALGA0088210 | *HJURP* | < 0.01 |
|  | 17 | 14.75-15.45 | INRA0052780 INRA0052808 | *BMP2***^*^** | < 0.001 |
| Front leg buck knee | 5 | 0.01- 0.04 | ASGA0102906 | *-* | < 0.001 |
|  | 6 | 7.23-7.77 | MARC0042729 MARC0012087  ASGA0093627 | *VAT1L NUDT7 MON1B* | < 0.001 |
|  | 6 | 10.14-10.38 | H3GA0056362 | *CALB2***^*^** *MARVELD3 TAT CHST4 PHLPP2* | < 0.01 |
|  | 11 | 12.14-12.73 | ALGA0060925 | *RFXAP SMAD9***^*^** *EXOSC8 FAM48A ALG5* | < 0.001 |
|  | 17 | 15.38-15.45 | MARC0070553 | *BMP2***^*^** | < 0.001 |
| Rear weak legs | 2 | 119.41 | MARC0086807 | *FBN2***^*^** *SLC27A6***^*^**  *ISOC1 ADAMTS19* | < 0.001 |
|  | 6 | 63.53 | ASGA0028870 | *MTF1 SF3A3 FHL3***^*^** *UTP11L POU3F1* | < 0.01 |
|  | 9 | 25.82-26.03 | ASGA0097582 MARC0090486 ASGA0097743 | *eEF1 MTNR1B***^*^**  *SLC36A4* | < 0.01 |
|  | 10 | 7.6 | DRGA001<0231 | *GPATCH2* | < 0.01 |
|  | 11 | 38.22 | H3GA0031835 | *-* | < 0.05 |
|  | 11 | 73.26 | ASGA0051894 | *-* | < 0.05 |
|  | 14 | 81.61 | H3GA0041090 | *COMTD1 ZNF503 C10orf41 C10orf11* | < 0.001 |
| Rear upright legs | 2 | 6.32 | ASGA0008834 | *FERMT3 CCDC88B MACROD1 FLRT1 OTUB1 COX8A* | < 0.001 |
|  | 4 | 15.05 | ASGA0085953 | *MTSS1***^*^** *NDUFB9***^*^**  *TMEM65 FER1L5 ANXA13* | < 0.01 |
|  | 4 | 110.71 | ASGA0021779 | *TRIM33 SYT6 OLFML3 HIPK1* | < 0.01 |
|  | 9 | 22.92-23.14 | ALGA0105323 ALGA0109892 | *NOX TRIM77 NAALAD2 CHORDC1* | < 0.001 |
|  | 9 | 107.27-107.35 | ALGA0054755 ASGA0044381 ASGA0044383 ASGA0044384 | *METTL13 DNM3* | < 0.001 |
|  | 10 | 43.47 | H3GA0030187 | *C1QL1 PTER* | <0.01 |
|  | 11 | 75.01-76.09 | ASGA0052016 M1GA0015368 LGA0063931 ASGA0052052 ASGA0052061 ASGA0084337 ALGA0063994 H3GA0032663 ASGA0052129 MARC0058566 | *EFNB2***^*^** *ARGLU1 FAM155A LIG4***^*^** *ABHD13 TNFSF13B***^*^** *MYO16***^*^** | < 0.001 |
|  | 12 | 17.19 | ALGA0065510 | *A4GTP3 TMEM101 MPP3* | < 0.05 |
|  | 16 | 67.71 | ASGA0095078 | *NMUR2***^*^** | < 0.01 |
|  | 16 | 75.5 | ASGA0099242 | *-* | < 0.01 |
|  | 17 | 9.55-9.59 | ASGA0075426 ALGA0093217 | *IDO1 ADAM18* | < 0.001 |
|  | 17 | 29.48 | ALGA0094162 | *-* | < 0.001 |
|  | 17 | 45.65 | ASGA0077119 | *TOP1 PLCG1* | < 0.01 |
|  | X | 91.39-91.84 | MARC0017716 ASGA0081243 | *-* | < 0.01 |
| Front legs turned in | 2 | 37.36 | MARC0021626 | *E2F8 ZDHHC13 LIM* | < 0.001 |
|  | 2 | 38.74-38.99 | ASGA0010196 M1GA0024370 ALGA0117101 | *KCNC1 MYOD1***^*^** *OTOG***^*^** *USH1C SUR1* | < 0.001 |
|  | 2 | 40.45 | MARC0112888 | *-* | < 0.01 |
|  | 4 | 7.91 | ASGA0018040 | *KCNQ3 OC90 EFR3A* | < 0.01 |
|  | 4 | 127.96 | ALGA0029261 | *GCLM DNTTIP2 BCAR3 FNBP1L DR1 TMED5* | < 0.001 |
|  | 6 | 0.14 | ALGA0120717 | *DBNDD1 AFG3L2 TUBB3 DEF8***^*^** | < 0.01 |
|  | 7 | 91.81-91.86 | ASGA0034758 MARC0075685 | *-* | < 0.001 |
|  | 9 | 98.64 | ALGA0054462 | *PUS7 RINT1 EFCAB10 ATXN7L1 NAMPT* | < 0.01 |
|  | 11 | 20.02 | ISU10000529 | *5HT2A ESTD LRCH1***^*^** | < 0.01 |
|  | 12 | 9.35 | ALGA0064929 | *MAP2K6***^*^** *ABCA5 ABCA6 ABCA9* | < 0.01 |
|  | 18 | 2.67 | ALGA0096804 | *HTR5A DPP6* | < 0.001 |
|  | 18 | 3.45-3.46 | ASGA0095228 MARC0044433 | *XRCC2* | < 0.001 |
| Rear legs turned in | 1 | 24.34 | ASGA0001646 | *VTA1 NMBR***^*^** | < 0.01 |
|  | 1 | 290.07 | MARC0111283 | *IER3IP1 KATNAL2 HDHD2 C18orf22 ADNP2* | < 0.1 |
|  | 2 | 124.38-124.41 | H3GA0007919 ALGA0016362 | *TCF7***^*^** *CDKL3 CDKN2AIPNL PHF15* | < 0.01 |
|  | 3 | 9.46 | ALGA0017453 | *CLDN4 CLIP2* | < 0.01 |
|  | 3 | 122.98 | ALGA0117199 | *-* | < 0.01 |
|  | 4 | 104.71 | H3GA0013785 | *PRKAB2***^*^** *PDE4DIP* | < 0.001 |
|  | 5 | 81.12 | ASGA0026618 | *TMPO* | < 0.001 |
|  | 6 | 101.95 | ALGA0037083 | *-* | < 0.001 |
|  | 8 | 15.75 | ASGA0038091 | *DHX15***^*^** *ANAPC4 SLC34A2***^*^** *RBPSUH TBC1D19* | < 0.05 |
|  | 11 | 54.45 | DRGA0011331 | *SLITRK1 SLITRK6* | < 0.001 |
|  | 11 | 73.88 | ASGA0051920 | *-* | < 0.001 |
|  | 12 | 8.28-8.75 | ASGA0053070 MARC0086052 H3GA0033435 ALGA0064900 | *IRK2 KCNJ16 MAP2K6***^*^** | < 0.001 |
|  | 14 | 146.49 | ASGA0068165 | *MGMT EBF3***^*^** *GLRX3 TCERG1L* | < 0.1 |
|  | 15 | 79.68-79.80 | MARC0009495 MARC0049797 | *CCDC141 MYBPH***^*^** *SESTD1* | < 0.001 |
|  | 15 | 80.35-80.60 | ASGA0102478 ALGA0114289  ASGA0070136 H3GA0044704 H3GA0044706 | *-* | < 0.001 |
|  | X | 0.83 | ASGA0090472 | *-* | < 0.01 |
|  | X | 7.23 | DRGA0017113 | *ARHGAP6 AMEL MSL3* | < 0.001 |
| Front feet size | 1 | 269.03 | ASGA0007035 | *-* | < 0.01 |
|  | 1 | 280.12 | M1GA0001575 | *STF1 NR6A1 PSB7 GPR144 OLFML2A WDR38 B5APV1 GOLGA1 RABEPK PPP6C* | < 0.01 |
|  | 1 | 283.69 | H3GA0005075 | *COQ4 FATP4 URM1 CERCAM ODF2 GLE1 SCOC SPTAN1 WDR34 SET PKN3***^*^** *ZDHHC12 ZER1 TBCD13 ENDOG***^*^** | < 0.001 |
|  | 10 | 48.22-48.34 | H3GA0030320 ALGA0059207 | *TSH1 PRTFDC1 ENKUR ARHGAP21***^*^** | < 0.001 |
|  | 11 | 56.66 | ALGA0062528 | *SLITRK1 SLITRK6 SLITRK5* | < 0.001 |
|  | 13 | 15.27 | ASGA0056454 | *STT3B OSBPL10* | < 0.001 |
|  | 14 | 2.77 | ALGA0074315 | *KSYK AUH* | < 0.001 |
|  | 15 | 14.82 | ALGA0084101 | *DARS UBXN4 LCT R3HDM1 ZRANB3 RAB3GAP1* | < 0.001 |
|  | 17 | 54.09 | INRA0054308 | *PTPN1***^*^** *PARD6B DPM1 MOCS3* | < 0.01 |
|  | 18 | 21.74-21.80 | ALGA0097487 ALGA0097488 | *SPAM1 HYAL4 WASL LMOD2 ASB15* | < 0.001 |
| Rear feet size | 2 | 37.03 | M1GA0002857 | *DBX1***^*^**  *NAV2 E2F8 ZDHHC13* | < 0.01 |
|  | 3 | 103.13-103.35 | ASGA0089483 | *ALK* **^*^** *FAM179A CLIP4 WDR43 TRMT61B* | < 0.01 |
|  | 5 | 4.35-5.00 | H3GA0015330 ALGA0030095 H3GA0015339 ASGA0024035 | *TNRC6B FAM83F GRAP2 ENTHD1 CACNA1I* **^*^** *RPS19BP1* | < 0.001 |
|  | 9 | 48.95-49.16 | MARC0112820 H3GA0027376 | *GRAMD1B ZNF202 OR6X1 OR6M1* | < 0.001 |
|  | 11 | 23.16-23.44 | ALGA0061496 ASGA0050421 ASGA0102583 | *DNAJC15 C13orf30 AKAP11* | < 0.01 |
|  | 17 | 47.23 | ALGA0095323 | *-* | < 0.01 |
|  | 17 | 48.99-49.23 | H3GA0049205 | *RIMS4 WISP2 YWHAB RABPC1L WFDC5 STK4* | < 0.01 |
| Front uneven toes | 4 | 130.05 | H3GA0014790 | *TGFBR3***^*^** *HFM1 ZNF644 BARHL2* | < 0.01 |
|  | 8 | 1.37 | H3GA0024036 | *-* | < 0.01 |
|  | 9 | 41.42 | H3GA0027118 | *-* | < 0.001 |
|  | 18 | 13.86 | ASGA0079021 | *-* | < 0.001 |
| Rear uneven toes | 6 | 74.52-74.71 | MARC0074797 | *OSBPL1A***^*^** *HRH4* | < 0.01 |
|  | 9 | 10.41-10.45 | ASGA0093058 ALGA0103116 ASGA0090332 | *PRKRIR C11orf30 LRRC32* | < 0.001 |
|  | 17 | 1.28-1.49 | ASGA0074962 MARC0072314 | *C8orf48* | < 0.001 |
|  | 17 | 3.49 | ALGA0092745 | *-* | < 0.001 |

^*^ The genes labeled with superscript asterisk indicate those potentially important ones relevant to skeleton development, bone and cartilage development, and energy metabolism using functional annotation through online DAVID (http://david.abcc.ncifcrf.gov/). P values indicate the significant candidate regions and were determined from bootstrap analysis based on the genetic variance of 5-SNPs sliding window.
